# Multinomial machine learning identifies independent biomarkers by integrated metabolic analysis of acute coronary syndrome

**DOI:** 10.1038/s41598-023-47783-5

**Published:** 2023-11-23

**Authors:** Meijiao Fu, Ruhua He, Zhihan Zhang, Fuqing Ma, Libo Shen, Yu Zhang, Mingyu Duan, Yameng Zhang, Yifan Wang, Li Zhu, Jun He

**Affiliations:** 1 Ningxia Medical University, Yinchuan, 750004 Ningxia China; 2 Department of Cardiology, General Hospital of Ningxia Medical University, Yinchuan, 750004 Ningxia China; 3 Department of Cardiology, Hanzhong Central Hospital, Hanzhong, 723200 Shanxi China; 4 Department of Cardiology, The Fifth People’s Hospital of Ningxia, Shizuishan, 753000 Ningxia China; 5 Center for Cardiovascular Diseases, People’s Hospital of Ningxia Hui Autonomous Region, Yinchuan, 750002 Ningxia China; 6 Department of Cardiology, The Second Affiliated Hospital of Henan University of Science and Technology, Luoyang, 471000 Henan China; 7 Department of Radiology, General Hospital of Ningxia Medical University, Yinchuan, 750004 Ningxia China

**Keywords:** Computational biology and bioinformatics, Molecular biology, Biomarkers, Cardiology, Risk factors

## Abstract

A multi-class classification model for acute coronary syndrome (ACS) remains to be constructed based on multi-fluid metabolomics. Major confounders may exert spurious effects on the relationship between metabolism and ACS. The study aims to identify an independent biomarker panel for the multiclassification of HC, UA, and AMI by integrating serum and urinary metabolomics. We performed a liquid chromatography-tandem mass spectrometry (LC–MS/MS)-based metabolomics study on 300 serum and urine samples from 44 patients with unstable angina (UA), 77 with acute myocardial infarction (AMI), and 29 healthy controls (HC). Multinomial machine learning approaches, including multinomial adaptive least absolute shrinkage and selection operator (LASSO) regression and random forest (RF), and assessment of the confounders were applied to integrate a multi-class classification biomarker panel for HC, UA and AMI. Different metabolic landscapes were portrayed during the transition from HC to UA and then to AMI. Glycerophospholipid metabolism and arginine biosynthesis were predominant during the progression from HC to UA and then to AMI. The multiclass metabolic diagnostic model (MDM) dependent on ACS, including 2-ketobutyric acid, LysoPC(18:2(9Z,12Z)), argininosuccinic acid, and cyclic GMP, demarcated HC, UA, and AMI, providing a C-index of 0.84 (HC vs. UA), 0.98 (HC vs. AMI), and 0.89 (UA vs. AMI). The diagnostic value of MDM largely derives from the contribution of 2-ketobutyric acid, and LysoPC(18:2(9Z,12Z)) in serum. Higher 2-ketobutyric acid and cyclic GMP levels were positively correlated with ACS risk and atherosclerosis plaque burden, while LysoPC(18:2(9Z,12Z)) and argininosuccinic acid showed the reverse relationship. An independent multiclass biomarker panel for HC, UA, and AMI was constructed using the multinomial machine learning methods based on serum and urinary metabolite signatures.

## Introduction

ACS, including UA and AMI, will occur as a result of a luminal thrombus or a sudden hemorrhage imposed on an atherosclerotic plaque^1, 2^. The thrombus is usually incomplete and dynamic or even absent in UA, whereas it is primarily occlusive and sustained in AMI, mainly caused by plaque rupture^3^. The formation and progression of atherosclerotic plaque is a complex process associated with atherosclerotic cardiovascular disease (ASCVD) events^4^. Perturbations in cardiac glucose, amino acid, and fatty acid metabolism are contributors to coronary atherosclerotic plaque and ACS pathologies^5^. Based on the different characteristics of plaque and clinical outcomes in different types of ACS, we speculate that UA and AMI exhibit specific small-molecule metabolite variations.

As a metabolism-related and multifactorial disease, ACS involves a complex interplay among aging, sex, weight, lifestyle, comorbidities, and adverse environmental exposures^6^. Metabolic phenotypes could be widely varied by gender, age, diet, physical activities, and other multifaceted factors^7–9^. Furthermore, these exogenous or endogenous confounders might exert some of their pathogenic effect on ACS via modification of the small metabolites^10^. Ultimately, the genuine disease signatures might be obscured or even masked by these confounders^11^. Therefore, in order to explore the genuine relationship between metabolites and ACS, some researchers call for adjustments for confounders that influence the host metabolome^12^. However, in the real-world recognition and classification problems, it is a tremendous challenge or even impossible to completely isolate confounders and disease-related metabolic features under the complex context of the human body.

The detailed small-molecule mechanism of the formation and progression of coronary atherosclerotic plaques in different types of ACS has not been uniformly concluded yet. The present studies are focused on the underlying small molecular activities and novel metabolic biomarkers related to the progression and severity of coronary artery disease (CAD)^13–16^. For recapitulating plaque formation, growth and rupture, researchers explored the plasma metabolomes of individuals with normal coronary artery (NCA), nonobstructive coronary atherosclerosis (NOCA), stable angina (SA), UA, and AMI^15^. Using a similar study design, the later report showed that N-acetyl-L-neuraminic acid (Neu5Ac) acted as a trigger for myocardial injury and accumulated progressively as CAD progressed^13^. Another two studies revealed that both the gut microbiota and metabolites changed significantly as CAD progressed, and a combined biomarker set may distinguish stable coronary artery disease (SCAD) from ACS^14, 16^. These metabolome analyses paid scant attention to major confounders of study outcomes, such as age, gender, and co-morbid conditions^17, 18^. In order to identify genuine disease-specific metabolome variance, the assessment and adjustment of the confounders are crucial for eliminating possible spurious effects on metabolism^12, 19, 20^.

Additionally, the studies mentioned above are multiclassification (N > 2) metabolomics studies that require us to map subjects into multiple categories^13–16^. Compared with binary classification (N = 2), multiclass omics is more intrinsically challenging in obtaining stable biomarkers^21, 22^. Although machine learning (ML) is widely used in metabolome classification, few ML algorithms are applied to construct multiclassification metabolomics models^23^. In order to convert the multiclassification into a binary classification, previous metabolomics studies on multiclassification of CAD usually conducted the multiple cross-comparisons based on hypothesis testing and then used binary classification ML approaches to obtain a classification model^15, 16^. For instance, LASSO regression usually failed to be used as a multinomial classifier in previous ACS metabolomic investigations^24, 25^. The multiclass metabolomics model for ACS and multinomial ML is lacking. A novel multiclass classification model for UA, AMI, and healthy controls using multinomial ML techniques is necessary.

In this study, we analyzed the serum and urine metabolic profiles from the 300 samples of the 150 participants through ultra-high liquid chromatography-tandem mass spectrometry (LC–MS/MS). Then, by performing the multinomial adaptive LASSO regression and RF classifier for multiclassification and adjusting for confounding factors, we developed a multiclass metabolite-based model that demarcated individuals with HC, UA or AMI. Our work may provide a power assist for metabolites to achieve early clinical translational applications for ACS.

## Materials and methods

### Study population and design

The graphical abstract of this study is illustrated in Supplemental Fig. S1. A total of 150 suspected ACS participants were consecutively enrolled and administered coronary angiography (CAG) at the General Hospital of Ningxia Medical University. All patients were confirmed with ≥ 50% reduction in luminal diameter by visual assessment. UA is defined as myocardial ischaemia at rest or on minimal exertion in the absence of acute cardiomyocyte injury/necrosis^26^. The diagnostic criteria for AMI need to be met by detecting the increase and/or decrease of cardiac biomarkers, preferably high sensitivity cardiac troponin (hs-cTn) T or I, with at least one value higher than the 99th percentile of the reference upper limit, and at least one of the following must be met: a. Symptoms of myocardial ischaemia; b. New ischemic ECG changes; c. Development of pathological Q waves on ECG; d. Imaging evidence of loss of viable myocardium or new regional wall motion abnormality in a pattern consistent with an ischemic etiology; e. Intracoronary thrombus detected on angiography or autopsy^26^. Subjects who showed no stenosis were regarded as healthy controls. Those with malignant tumors, autoimmune disorders, infectious diseases, and severe renal dysfunction with creatinine > 3.0 mg/dl were excluded. The Ethics Review Committee of the General Hospital of Ningxia Medical University authorized this study, which followed the Declaration of Helsinki’s guidelines. Written informed consent was received from all the participants before the study launched.

### Sample size calculation

We performed power analysis using MetaboAnalyst 5.0. The power reaches an acceptable level (0.8) at a sample size of approximately 120 (per group ≈ 40, Supplementary Fig. S1). If power is set at 0.75, the sample size is approximately 90 (per group ≈ 30, Supplementary Fig. S2). We set the sample size to 150, which meets the sample size standard. The detailed procedure for sample size calculation was provided in the Supplementary Fig. S1.

### Quantification of plaque burden

The SYNTAX score and modified Gensini score were applied to evaluate the severity of ACS. The SYNTAX score was used to grade the complexity of coronary lesions^27^. The modified Gensini score was used to quantify the atherosclerotic plaque burden of ACS, considering the location, number, and degree of stenosis^28^. The SYNTAX score I was calculated using an online pre-defined algorithm named the SYNTAX score calculator version 2.11 (http://www.syntaxscore.com/). The SYNTAX score II (http://www.syntaxscore.com/calculator/syntaxscore/framesetss2.htm) was computed by the SYNTAX score I, unprotected left main CAD; other clinical variables included age, sex, left ventricular ejection fraction, creatinine clearance, chronic obstructive pulmonary disease, and peripheral vascular disease. The modified Gensini scores take the severity score, the region multiplying factor, and the collaterals with the severity score adjustment factor into consideration^28^.

### Sample preparation

The metabolomics workflow complies with the published guidelines^29–31^. Paired morning whole blood and urine samples were collected from all subjects before CAG and centrifuged at 3000 rpm for 10 min at 4 °C. Serum and the urine supernatant were kept and aliquoted, respectively, and then stored at − 80 °C immediately for metabolic analysis. For the metabolite extraction, 50 μL of the samples were transferred to an Eppendorf tube. After the addition of 200 μL extract solution (acetonitrile: methanoll = 1:1, containing an isotopically labeled internal standard mixture), the samples were vortexed for 30 s, sonicated for 10 min in an ice-water bath, and incubated for 1 h at − 40 °C to precipitate proteins. Then the samples were centrifuged at 12,000 rpm (RCF = 13,800 g, R = 8.6 cm) for 15 min at 4 °C. The resulting supernatants were transferred to a fresh glass vial for analysis. The quality control (QC) samples were prepared by mixing an equal aliquot of the supernatants from all of the samples. One QC sample was inserted in every 10 test samples to monitor the repeatability of the analysis process.

### Untargeted metabolomics detection by ultra high-performance liquid chromatography/quadrupole exactive–orbitrap mass spectrometry (UHPLC/QE–MS)

A UHPLC system (Vanquish, Thermo Fisher Scientific) with a UHPLC BEH Amide column (2.1 mm × 100 mm, 1.7 μm) coupled to QE HFX MS (Orbitrap MS, Thermo) was performed in both positive and negative ionization modes. A mixture of 25 mmol/L ammonium acetate and 25 mmol/L ammonia hydroxide in water (pH = 9.75) (A) and acetonitrile (B) made up the mobile phase. The auto-sampler temperature was 4 °C, and the injection volume was 2 μL. For its capacity to acquire MS/MS spectra in information-dependent acquisition (IDA) mode under the supervision of the acquisition software (Xcalibur, Thermo), the QE HFX mass spectrometer was utilized. The acquisition software continuously assessed the full scan MS spectrum in this mode. The ESI source criteria were established: the capillary temperature was 350 °C, the sheath gas flow rate was 30 Arb, the auxiliary gas flow rate was 25 Arb, the full MS resolution was 60,000, the MS/MS resolution was 7500, the collision energy was 10/30/60 in NCE mode, and the spray voltage was either 3.6 kV (positive) or − 3.2 kV (negative), as appropriate.

### Metabolomics data preprocessing and assessment of the data quality

The criteria for assessing the quality of metabolomics data, the stability and reproducibility of the experimental method are as follows: the tolerance limits are set such that the measured response detected in two-thirds of QC samples is within 30% coefficient of variation^29, 31^. In this study, the internal standard of relative standard deviation (RSD) in QC samples was ≤ 15% (median), which represents high data quality. The raw data were imported to ProteoWizard and processed with R and XCMS for peak detection, extraction, alignment, and integration. Then, after obtaining the ion intensities for each peak, we created a matrix with the names of the samples, retention time-m/z pairings, and peak intensities.

The deviation value is filtered based on the RSD ≥ 30%^29, 31^. By eliminating peaks with missing values in more than 50% of samples, the matrix was further condensed^29, 31^. The residual missing value was filled up by one-half of the minimum value. Each retained peak was normalized using an internal standard.

### Metabolite identification

The molecular mass data (m/z) were aligned to identify metabolites using our in-house metabolite library, and public databases including the Kyoto Encyclopedia of Genes and Genomes databases (KEGG) (http://www.genome.jp/kegg/), Human Metabolome Database (HMDB) (http://www.hmdb.ca), and Metabolite Link (METLIN) (https://metlin.scripps.edu). The compound matching in this study is qualitative based on the dual-core algorithm (the dot-product function and the Euclidean distance)^32^. The authentication accuracy of the algorithms exceeds 70%^32^. Known metabolites reported in this study conformed to confidence level 1 (the highest confidence level of identification) of the Metabolomics Standards Initiative^33, 34^.

### Pathway analysis

We used MetaboAnalyst 5.0 (https://www.metaboanalyst.ca/MetaboAnalyst/) to perform pathway topology analysis based on KEGG databases. The statistical significance of the changes in pathways was evaluated by the Hypergeometric test, the default method used by MetaboAnalyst 5.0. The topological pathway impacts were quantified using the published method^35^. Using the K-means cluster method to observe the change trend of metabolites.

### Feature selection

The adaptive LASSO regression was one of the most robust machine learning approaches for feature selection and classification. To recognize robust metabolites to simultaneously discriminate HC, UA, and AMI, the adaptive LASSO multinomial regression was performed using the ‘cv. glmnet’ package. The overfitting risk of classifier was rendered through tenfold internal cross-validation. The optimal features were captured at the minimum λ with adaptive multinomial LASSO regression. Then, random forest (RF), another ensemble machine-learning approach for classification, was applied. The ‘createDataPartition’ function in the ‘caret’ package was applied to randomly divide the data into a 75% traininging set and a 25% test set. The ‘randomForest’ package yielded lists of metabolites sorted by feature importance. The optimal number of discriminant metabolites was identified using tenfold cross-validation implemented with the “rfcv” function in the R package ‘randomForest’ with five repeats. The effectiveness of machine learning algorithms was displayed by confusion matrices and multi-group receiver operating characteristic (ROC) curves. Multi-group ROC curves were displayed by ‘multiclass. Roc’ in the ‘pROC’ package. To improve reproducibility and model robustness, the 28 shared metabolic features (15 in serum and 13 in urine) with the two algorithms were selected as candidate metabolic biomarkers. The intersection of variables in the two machine learning approaches was visualized as a Venn diagram by the ‘VennDiagram’ package.

### Simplification of features with subgroup interaction test

In order to explore the genuine relationship between metabolites and ACS, the multivariable-adjusted model and subgroup interaction test were used to recognize metabolites that were not roiled by confounders among the 28 candidate metabolites. The ‘mgcv’ package was used to conduct stratified and interaction analyses for exploring the associations between the metabolic signatures and ACS risk in different subgroups, such as different age, sex, BMI, smoking status, history of hypertension or diabetes, or levels of TG, TC, HDL-C, and LDL-C. The forest plot of univariable and multivariable-adjusted models was visualized by the ‘ggforestplot’ package, and the forest plot of subgroup interaction was depicted by the ‘forestploter’ package.

### Construction of the multiclass metabolic diagnostic model

The multiclass metabolic diagnostic model (MDM) was developed via multivariate generalized linear regression (R package ‘glm’). The contribution of each metabolite to the MDM is calculated by the ‘calc.relimp’ function in the ‘relaimpo’ package. The jitter plot of the cutoff is using the ‘ggsignif’ and ‘ggplot2’ packages. Consistency between actual and integrated model-predicted probabilities and the overfitting risk of MDM was assessed using the calibration curve (1000 resampling bootstraps) in the internal validation set. Hosmer–Lemeshow p > 0.05 reveals good consistency between actual and predicted probabilities, and MDM is not overfitted. The clinical application of alternative diagnostic strategies was determined with decision curve analysis (DCA) by quantifying the net benefits at various threshold probabilities.

### Internal validation of the multiclass metabolic diagnostic model

The discrimination ability of the integrated model was measured using the Harrell concordance index (C-index) with 1000 resampling bootstraps in internal validation. The Net Reclassification Index (NRI) was employed to compare the diagnostic value of a single metabolite with the combined metabolic panel.

### Statistical analysis

**Figure 1:**
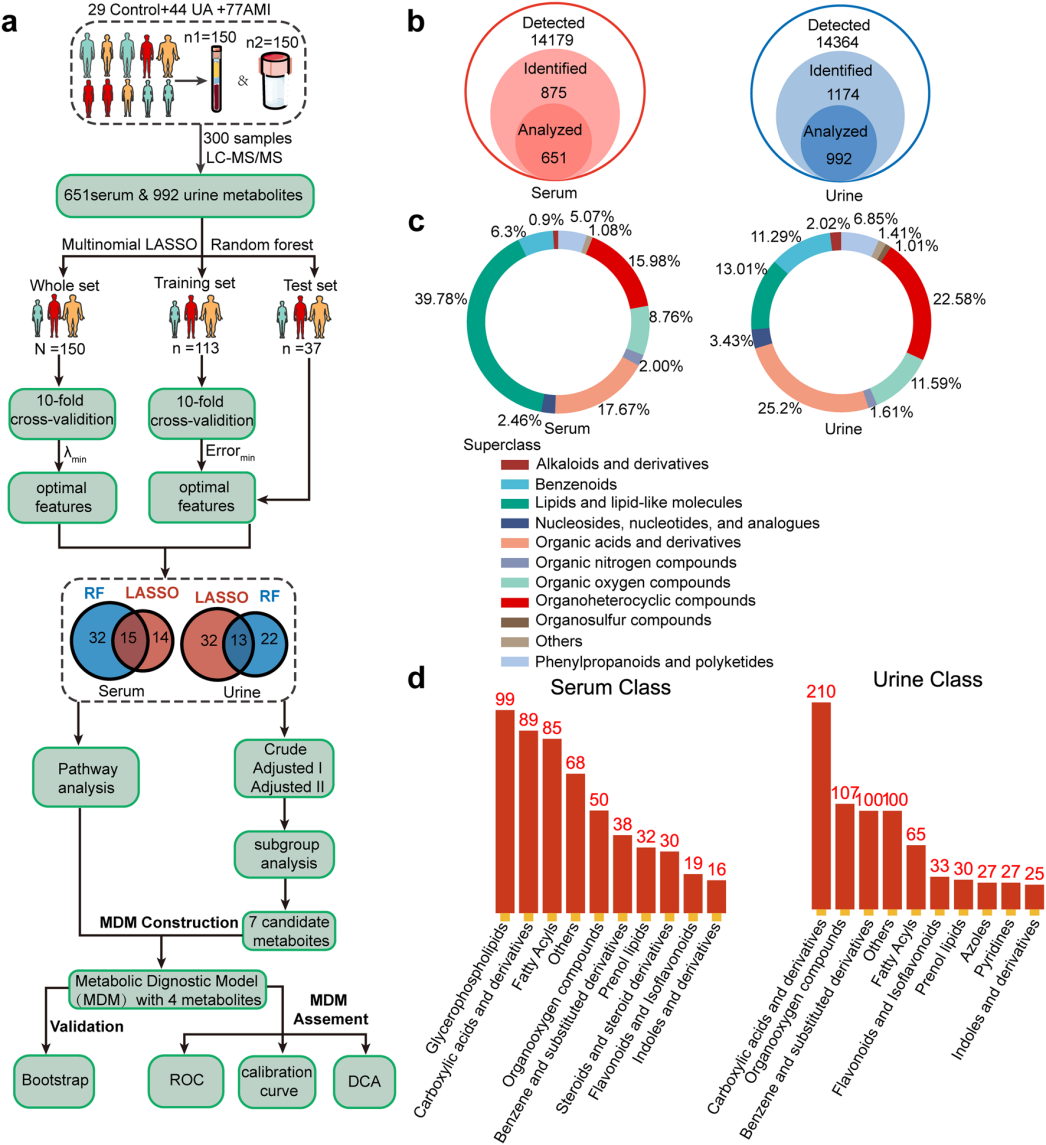
Overview of metabolome detection in serum and urine. (**a**) A schematic summarizing the workflow for statistical analysis. (**b**) Total count of serum metabolites and urinary metabolites. (**c**) Circular diagram of superclass composition in serum metabolites and urinary metabolites. (**d**) The top 10 detected classes and the count of compounds contained in each class of serum and urine.

R 4.2.1 was used for data analysis and visualization. The workflow of statistical analysis is presented in Fig. 1a. The differences among baseline characters were measured by (1) continuous normal distribution variables among three groups were analyzed by a one-way analysis of variance. The Kruskal–Wallis H-test was applied for data not distributed normally. (2) Continuous, normally distributed variables between two groups were analyzed by the Student’s *t*-test. The Mann–Whitney U test was applied to data that was not normally distributed. (3) Categorical variables were compared by the χ^2^ test. The Kruskal–Wallis H-test was used to compare the metabolites' intensity between the three groups. The relationship between metabolites and the ACS phenotype, as well as the relationship between serum and urine metabolites were expressed by the Spearman correlation analysis. All the tests were two-sided, and *p* < 0.05 indicates significance unless otherwise stated. The Benjamin-Hochberg correction based on false discovery rate (FDR) was utilized in multiple tests to decrease false-positive rates, and adjusted *p* < 0.05 indicates significance.

### Ethics approval and consent to participate

The study was approved by the Ethics Committee for the Conduct of Human Research at the General Hospital of Ningxia Medical University (2020-763). All participants were informed of the possible risks of the study and gave written informed consent. All methods were carried out in accordance with relevant guidelines and regulations.

## Results

### General characteristics of the enrolled population

As shown in Table 1, the UA and the AMI are older than the HC (HC vs. UA, adjusted *p* ˂ 0.05; HC vs. AMI, adjusted *p* ˂ 0.05), and traditional cardiovascular risk factors, such as smoking, hypertension, diabetes mellitus, and hyperlipidemia, are much more frequently presented compared with the HC (adjusted *p* ˂ 0.05). The AMI has higher levels of total cholesterol (TC) and low-density lipoprotein cholesterol (LDL-C) than the UA with disease shifting (adjusted *p* ˂ 0.05). The UA and AMI display lower red blood cell (RBC) counts and hemoglobin (HGB) levels compared with the HC group (adjusted *p* ˂ 0.05). The white blood cell (WBC) and neutrophil (NEUT) counts and hypersensitive C-reactive protein (hs-CRP) levels in the AMI group are significantly increased compared with the HC and the UA (adjusted *p* ˂ 0.05). With the progress of the disease, levels of aspartate transaminase (AST), alanine aminotransferase (ALT), and Recombinant N-terminal Pro-Brain Natriuretic peptide (NT-proBNP) are increased (adjusted *p* ˂ 0.05). The AMI exhibits higher SYNTAX I, Gensini scores, number of stenosed vessels (No. of SV), and cTnI levels, as well as lower LVEF compared with the UA (adjusted *p* ˂ 0.05). Based on the above, subjects suffering from AMI experienced the most stressful inflammation, metabolomic disorders, the most severe coronary artery lesion, and left ventricular dysfunction.

**Table 1:** Baseline characteristics of the enrolled population.

	HC (n = 29)	UA (n = 44)	AMI (n = 77)	p value
Age (years)^†^	49.97 ± 5.21	61.00 ± 8.15	59.05 ± 9.29	< 0.001^ab^
Male^§^	25 (86.21)	25 (56.82)	59 (76.62)	0.01^ac^
BMI, kg/m^2†^	24.90 ± 2.62	25.17 ± 3.14	24.59 ± 2.75	0.55
Current smoker^§^	25 (86.21)	18 (40.91)	40 (51.95)	< 0.001^ab^
CAD family history^§^	0 (0.00)	9 (20.45)	7 (9.09)	0.02^a^
Hypertension^§^	0 (0.00)	23 (52.27)	35 (45.45)	< 0.001^ab^
Diabetes mellitus^§^	0 (0.00)	17 (38.64)	19 (24.68)	< 0.001^ab^
Hyperlipidemia^§^	0 (0.00)	7 (15.91)	35 (45.45)	< 0.001^abc^
Laboratory data				
WBC (*10^9^/L)^†^	6.64 ± 1.57	6.68 ± 2.05	9.88 ± 2.88	< 0.001^bc^
NEUT (*10^9^/L)^†^	3.68 ± 1.25	3.97 ± 1.86	7.25 ± 2.79	< 0.001^bc^
LYM (*10^9^/L)^†^	2.17 ± 0.63	2.03 ± 0.53	1.87 ± 0.80	0.13
RBC (*10^12^/L)^†^	5.04 ± 0.48	4.66 ± 0.41	4.69 ± 0.57	< 0.01^ab^
HGB (g/L)^†^	159.45 ± 13.76	141.18 ± 16.52	142.86 ± 19.15	< 0.001^ab^
PLT (*10^9^/L)^†^	226.86 ± 55.38	229.36 ± 61.67	243.29 ± 73.59	0.39
BUN (mmol/L)^†^	4.72 ± 1.07	5.23 ± 1.59	5.40 ± 1.57	0.12
Scr (μmol/L)^†^	68.32 ± 9.65	62.91 ± 12.86	70.86 ± 18.32	0.03^c^
UA (μmol/L)^†^	320.97 ± 62.35	308.16 ± 71.91	324.64 ± 86.07	0.53
eGFR (ml/min/1.73m^2^)^†^	110.0 ± 12.7	116.5 ± 27.8	108.8 ± 27.3	0.26
Ccr (mL/min)^†^	114.01 ± 20.11	105.17 ± 27.31	100.98 ± 28.13	0.08
Glucose (mmol/L)^†^	4.97 ± 0.38	6.36 ± 1.68	6.99 ± 2.35	< 0.001^ab^
AST (U/L)*	19.00 (16.70–21.90)	23.20 (20.80–29.07)	56.60 (32.80–131.20)	< 0.001^abc^
ALT (U/L)*	21.60 (17.10–24.30)	28.00 (20.28–36.75)	37.70 (29.40–53.10)	0.02^abc^
TG (mmol/L)^†^	1.70 ± 0.85	1.89 ± 1.10	1.71 ± 0.74	0.53
TC (mmol/L)^†^	4.32 ± 0.82	3.49 ± 0.77	4.09 ± 1.01	< 0.001^ac^
HDL-C (mmol/L)^†^	1.24 ± 0.29	0.96 ± 0.18	0.91 ± 0.23	< 0.001^ab^
LDL-C (mmol/L)^†^	2.36 ± 0.60	1.80 ± 0.67	2.58 ± 0.83	< 0.001^ac^
HCY (μmmol/L)^†^	20.92 ± 10.10	22.19 ± 16.72	24.33 ± 17.89	0.58
hs-CRP (mg/L)*	0.47 (0.21–0.71)	1.34 (0.36–2.51)	6.92 (3.01–20.77)	< 0.001^bc^
cTnI (ng/ml)*	0.01 (0.01–0.01)	0.01 (0.01–0.01)	6.93 (1.34–20.10)	< 0.001^bc^
NT-proBNP (pg/ml)*	49.46 (36.19–115.10)	107.52(52.71–281.05)	858.00(325.59–1788.50)	< 0.001^abc^
SYNTAX score I*	NA	15.50 (9.75–20.00)	17.00 (12.00–26.50)	< 0.01^c^
SYNTAX score II*	NA	23.05 (20.12–28.93)	22.80 (19.95–30.70)	0.48
Gensini score*	NA	42.50 (28.25–69.50)	57.00 (39.50–74.50)	0.03 ^c^
No. of stenosed vessels^§^				< 0.001^abc^
None	29 (100.0)	0 (0.0)	0 (0.0)	
One	0 (0.0)	9 (20.5)	24 (31.2)	
Two	0 (0.0)	14 (31.8)	21 (27.3)	
Three	0 (0.0)	21 (47.7)	32 (41.6)	
LVEF (%)^†^	67.2 ± 2.7	64.3 ± 8.3	52.9 ± 8.3	< 0.001^bc^

^†^Mean ± SD, *median (IQR), ^§^n (%), adjusted *P*^a^ < 0.05 for equality between HC vs. UA. adjusted *P*^b^ < 0.05 for equality between HC vs. AMI. adjusted *P*^c^ < 0.05 for equality between UA vs. AMI.

### Machine learning for identifying serum multiclass diagnostic metabolites

**Figure 2:**
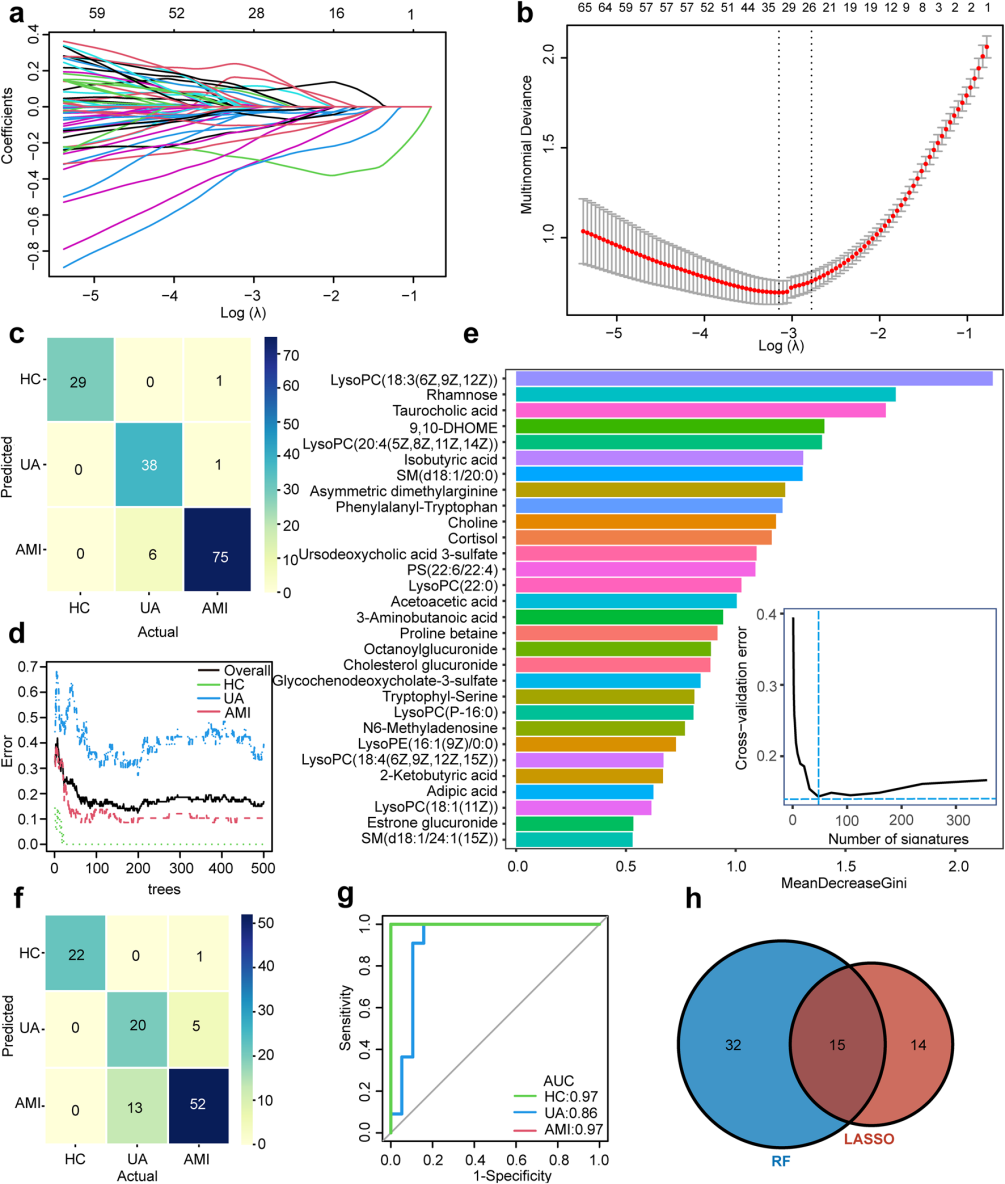
The 15 candidate serum metabolic biomarkers selected by adaptive LASSO multinomial regression and random forest algorithms. (**a**) Plots for adaptive LASSO multinomial regression coefficients over different values of the penalty parameter λ. (**b**) Cross-validation plots for the penalty parameter λ. The dashed line left represents the minimum λ. The 29 candidate metabolic signatures mapping the minimum λ (0.0.4) were subjected to the next analysis. (**c**) The confusion matrix of the internal cross-validation set shows 29 HC, 38 UA, and 75 AMI are correctly classified by the adaptive LASSO multinomial algorithm. The darker the color represents, the more correctly it is classified. (**d**) The correlation plots between the number of random forest trees and the model classification error. The error stabilized when using 500 trees. (**e**) The top 30 discriminant metabolic signatures are ranked in descending order of importance to the accuracy of the RF classifier for HC, UA, and AMI. The bar lengths indicate the importance of the signature. The insert represents a tenfold cross-validation error as a function of the optimal number of input signatures used to fit the RF classifier. The number of signatures against the cross-validation error curve reaches the inflection point when using 47 signatures. (**f**) The confusion matrix of the training set (n = 113) based RF classifier shows 94 subjects are correctly classified. (**g**) The multinomial receiver-operating characteristic (ROC) curves are used to distinguish HC, UA, and AMI in the internal test set (n = 37) based RF classifier. (**h**) The Venn diagram shows the shared 15 candidate serum biomarkers selected by adaptive LASSO multinomial regression and RF algorithms.

In serum samples, a total of 14,179 features were profiled in both positive and negative electrospray ionization (ESI^+^ and ESI^−^) modes. Based on KEGG and HMDB analyses, 651 metabolites were identified after peak alignment and data preprocessing (Fig. 1b). The lipids and lipid-like molecules were the most abundant, of which glycerophospholipids ranked first (Fig. 1c,d). The adaptive LASSO multinomial regression and random forest, the branches of artificial intelligence, were used to reduce our extensive metabolites to a small set of candidate diagnostic biomarkers for discriminating the multiple groups. To more comprehensively mine the metabolite information without omission, we fed all the identified 651 serum metabolites into the adaptive LASSO multinomial regression classifier for variable selection. We utilized ten-fold cross-validation to select the penalty parameter λ. The variables included in the adaptive LASSO multinomial regression and their corresponding coefficients for the different values of λ are presented in Fig. 2a. As the penalty parameter λ increases, the variable coefficients are forced to zero. We adopted a minimum λ (λ _min_ = 0.04) to fit the LASSO regression (Fig. 2b). Then we identified 29 key serum metabolites with nonzero coefficients, demarcating HC, UA, and AMI (Fig. 2b). The 29 metabolites were considered candidate biomarkers for further analysis. The effectiveness of the LASSO classifier is presented by a confusion matrix in that total 142 subjects are correctly classified, and the correct classification rate is 94.7% (Fig. 2c). We further conducted internal cross-validation to verify whether the model is overfitted. The classifier is not overfitted with 85.3% of the correct classification rate, calculated via the ‘cv. glmnet’ package. Meanwhile, the RF algorithm was applied to select the optimal features for multiclass HC, UA, and AMI. Based on the correlation plot between the number of RF trees and RF model error, the number of trees against the error curve tended to stabilize when 500 trees were chosen as the final model’s parameter (Fig. 2d). The minimum cross-validation error was obtained when using 47 important metabolites in the number of signatures against the cross-validation error curve (Fig. 2e). The top 30 discriminant metabolites are displayed in Fig. 2e. In the training set (n = 113), a sum of 94 subjects was classified precisely, and the correct classification rate was 83.2% (Fig. 2f). In the internal test set (n = 37), the AUC of this RF classifier to distinguish the HC, UA, and AMI was calculated as 0.97, 0.86, and 0.97, respectively, by the multiclass ROC analysis (Fig. 2g). For more robust biomarker identification, we adopted the intersection of the two algorithms and obtained 15 shared candidate diagnostic signatures (Fig. 2h).

### Machine learning for identifying urinary multiclass diagnostic metabolites

A total of 14,364 metabolites in urine samples were detected, and 992 metabolites were analyzed (Fig. 1b). The organic acids and derivatives were the most abundant (Fig. 1c). We performed the two multinomial machine learning algorithms to obtain key urinary metabolites for distinguishing the three groups from each other. The adaptive LASSO multinomial regression captured 45 urinary candidate metabolites when λ _min_ = 0.03 (Supplemental Fig. S3a,b). The fitted LASSO classifier displayed a 95.3% correct rate overall (Supplemental Fig. S3c) and an 81.3% correct rate in the internal cross-validation. This proves that the multinomial LASSO classifier is not overfitted. Based on 500 trees, the RF classifier was constructed, and the number of signatures against the cross-validation error curve reached the nadir inflection point when using 35 important metabolites (Supplemental Fig. S3e). The top 30 discriminant metabolites are ranked by importance (Supplemental Fig. S3e). In the training set (n = 113), the correct classification rate of this RF algorithm was 72.6% (Supplemental Fig. S3f). In the internal test set (n = 37), the AUCs of this RF classifier were 0.81, 0.78, and 0.83 for distinguishing the HC, UA, and AMI, respectively (Supplemental Fig. S3g). Then, thirteen shared metabolites were identified by the two algorithms in a Veen diagram (Supplemental Fig. S3h).

### Altered serum and urine metabolic signatures and pathways as ACS progressed

**Figure 3:**
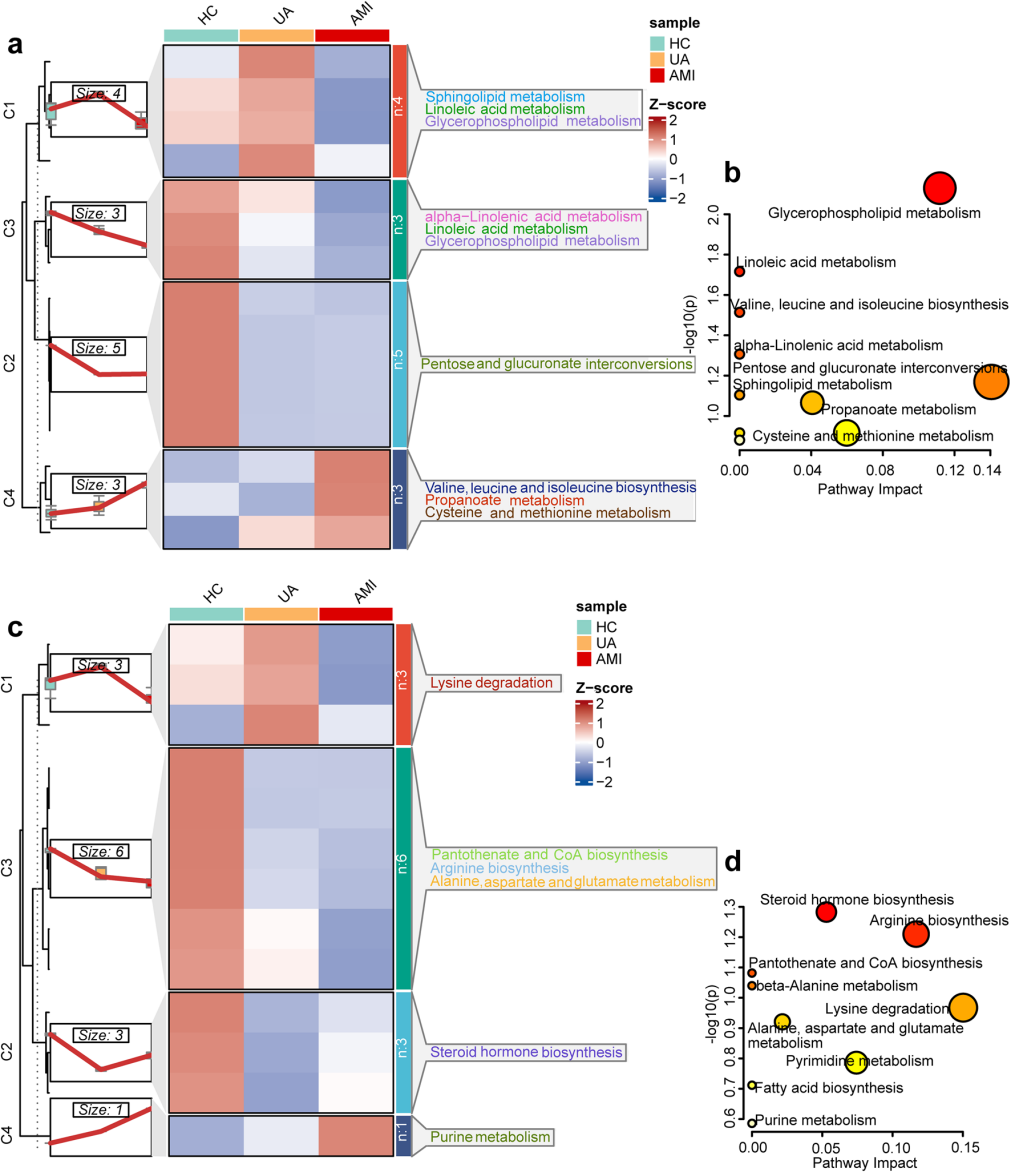
Altered metabolites and metabolic pathways as ACS progresses. (**a**,**c**) K-means clustering of the 15 serum and 13 urine metabolic signatures during HC, UA, and AMI. Different changing trends and major pathways of serum or urine metabolites are summarized in cluster 1–4. (**b**,**d**) Metabolic pathways undergo significant changes during HC, UA, and AMI in serum and urine.

**Figure 4:**
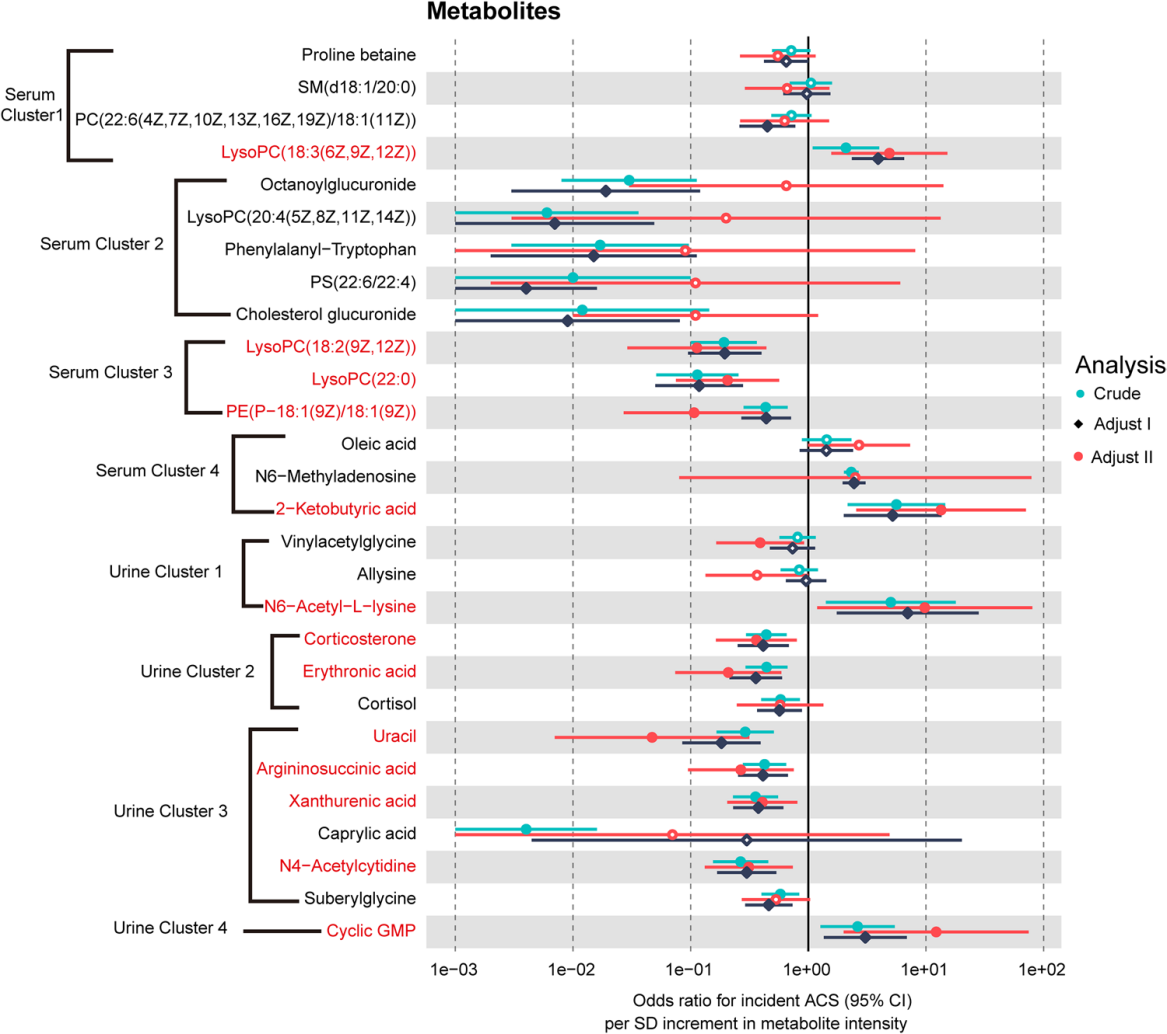
Univariable and multivariable analysis of the association between the candidate metabolic signatures and ACS. Crude (unadjusted OR); Adjust I (age, sex, BMI); Adjust II (age, sex, BMI, TG, TC, HDL-C, LDL-C, hypertension, diabetes, and smoking). The non-significant entries were drawn as hollow points. p < 0.05 was considered statistically significant.

We have obtained 15 and 13 candidate metabolic diagnostic signatures to multiclass HC, UA, and AMI in serum and urine samples. To explore metabolic alterations during ACS development, we examined metabolic trends applying the K-means clustering method to the candidate metabolic diagnostic signatures. The 15 serum discriminate metabolites were divided into four types of clusters. Specifically, cluster 3 and cluster 4 showed regular changes (Fig. 3a). Three metabolites, such as 2-ketobutyric acid in cluster 4 displayed a sharp increase in the transition of HC → UA → AMI (Figs. 3a, 4). The topological pathway analysis revealed that valine, leucine and isoleucine biosynthesis, cysteine and methionine metabolism were disturbed in cluster 4 during HC → UA → AMI (Fig. 3a,b). Cluster 3 including LysoPC(18:2(9Z,12Z)), LysoPC(22:0), and PE(P-18:1(9Z)/18:1(9Z)) were dropped step by step as ACS progressed (Figs. 3a, 4). Glycerophospholipid metabolism was the most significant disturbed pathway in cluster 3 during HC → UA → AMI (Fig. 3a,b). In urine samples, the 13 discriminate metabolites were clustered into 4 groups, cluster 3 and 4 showed regular alterations (Fig. 3c). The six metabolites, such as argininosuccinic acid in cluster 3 displayed a downward trend during HC → UA → AMI. The topological pathway analysis revealed that arginine biosynthesis was the most important pathway mapping cluster 3 during HC → UA → AMI (Fig. 3c,d). Cluster 4 including cyclic GMP demonstrated an upward trend from the HC to UA and then to the AMI and was enriched in purine metabolism (Figs. 3c,d, 4).

### Identification of independent metabolic signatures

In order to discover robust biomarkers that are independent of conventional cardiovascular risk factors, we conducted logistic regression analyses and interaction tests. The odds ratio (OR) per standard deviation (SD) of the 15 candidates in serum and the 13 candidates in urine are depicted in Fig. 4 and Supplemental Table S1, including the crude model (i.e., the unadjusted model), the parsimonious model (i.e., Adjust I, a minimally adjusted model that includes the covariates age, sex, and BMI), and the fully adjusted model (i.e., Adjust II, a model that includes the covariates age, sex, BMI, triglyceride [TG], total cholesterol [TC], high-density lipoprotein cholesterol [HDL-C], low-density lipoprotein cholesterol [LDL-C], hypertension, diabetes, and smoking). In the crude model, 11 metabolites in serum and 11 metabolites in urine are associated with ACS. After adjusting for various variables, a total of 5 serum metabolites, including 1 metabolite in serum cluster 1, serum cluster 3, and 1 metabolite in serum cluster 4, remained statistically significant (*p* < 0.05). Eight urine metabolites remained statistically significant after adjusting for various variables (*p* < 0.05). Each SD of LysoPC(18:3(6Z,9Z,12Z)), 2-ketobutyric acid, N6-Acetyl-l-lysine, and cyclic GMP was associated with a 3.89-fold (95% CI 1.57–15.23), 12.44-fold (95% CI 2.56–70.69), 8.77-fold (95% CI 1.19–80.26), and 11.22-fold (95% CI 2.00–74.71) increment in the OR for incident ACS. The risk of suffering from ACS was reduced by 0.89-fold (95% CI 0.03–0.45), 0.79-fold (95% CI 0.08–0.57), 0.89-fold (95% CI 0.03–0.42), 0.64-fold (95% CI 0.16–0.80), 0.79-fold (95% CI 0.07–0.59), 0.95-fold (95% CI 0.01–0.31), 0.73-fold (95% CI 0.10–0.76), 0.59-fold (95% CI 0.21–0.81), and 0.69-fold (95% CI 0.13–0.74) for each SD increase of metabolites in LysoPC(18:2(9Z,12Z)), LysoPC(22:0), PE(P-18:1(9Z)/18:1(9Z)), corticosterone, erythronic acid, uracil, argininosuccinic acid, xanthurenic acid, and N4-Acetylcytidine, respectively.

**Figure 5:**
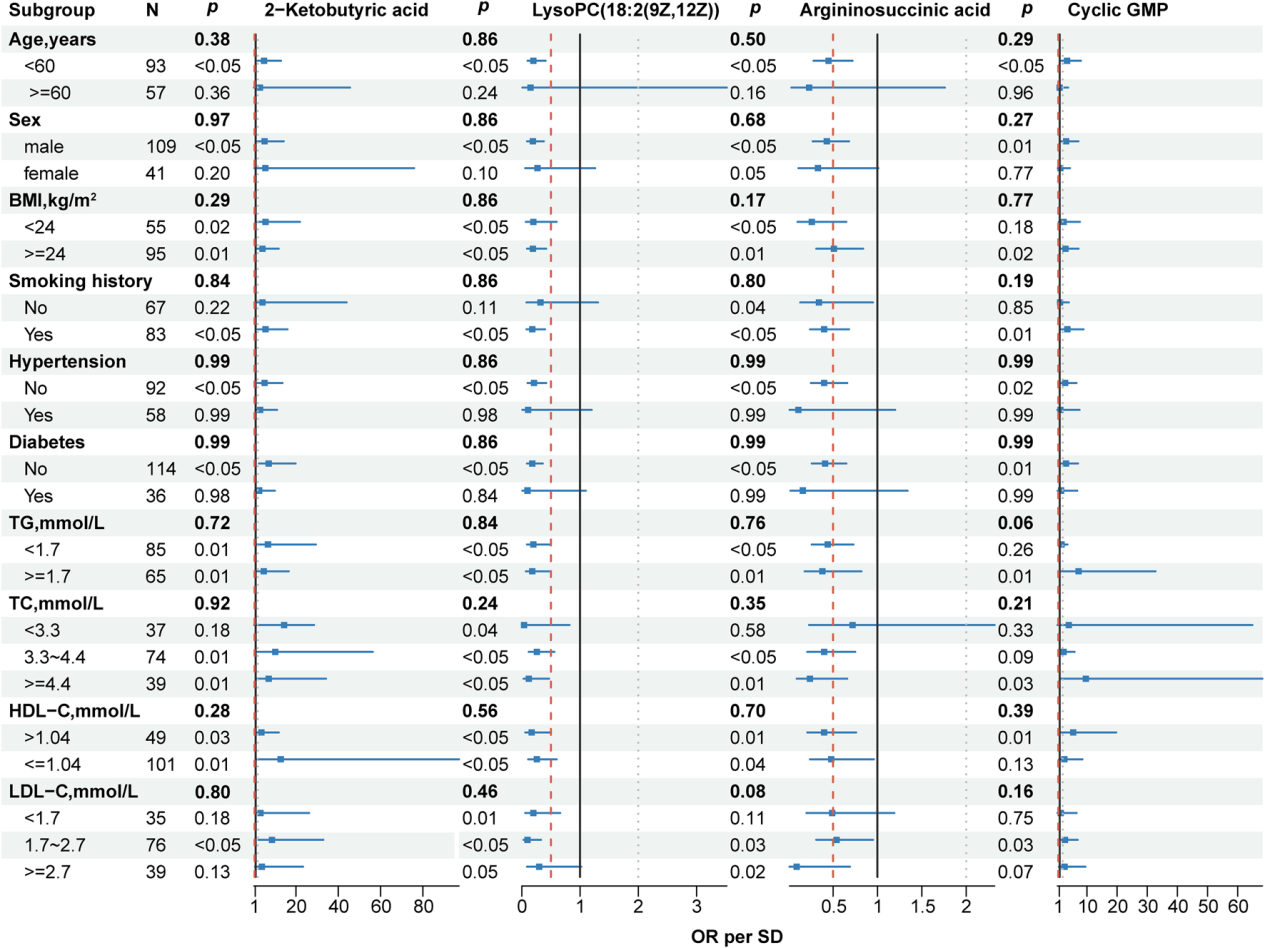
Subgroup analysis of 2-ketobutyric acid, LysoPC(18:2(9Z,12Z)), argininosuccinic acid, and cyclic GMP. The *p* for interaction revealed that age, gender, BMI, smoking history, hypertension, diabetes, TG, TC, HDL-C, and LDL-C played no interactive role in the association between the four metabolites and ACS. The *p* for interaction were drawn as bold in p entries. p < 0.05 was considered statistically significant. *OR per SD* per-standard deviation odds ratio.

Then subgroup analysis and interaction tests were carried out for the selected thirteen metabolites (Fig. 5, Supplemental Figs. S4–S6). Subgroup analysis revealed that the relationship between 2-ketobutyric acid and ACS remained consistent across subgroups of BMI, TG, and HDL-C (*p* for interaction > 0.05, Fig. 5). Although the same relationship was not observed in the ≥ 60 y, females, non-smokers, subjects with hypertension or diabetes, or subjects with different levels of TC and LDL-C, the interaction term indicated insignificant effect modification by subgroup variables for the association between 2-ketobutyric acid and ACS (*p* for interaction > 0.05, Fig. 5). Meanwhile, we noticed that subgroup variables played no interactive role in the relationship between the other six metabolites: LysoPC(18:2(9Z,12Z)), argininosuccinic acid, cyclic GMP, xanthurenic acid, erythronic acid, and N6-Acetyl-L-lysine and ACS (*p* for interaction > 0.05, Fig. 5, Supplemental Figs. S4–S6). Seven molecules out of the thirteen metabolites were not affected by those confounding factors and had the potential to be used to characterize ACS.

### Correlations between metabolic signatures and ACS phenotype

**Figure 6:**
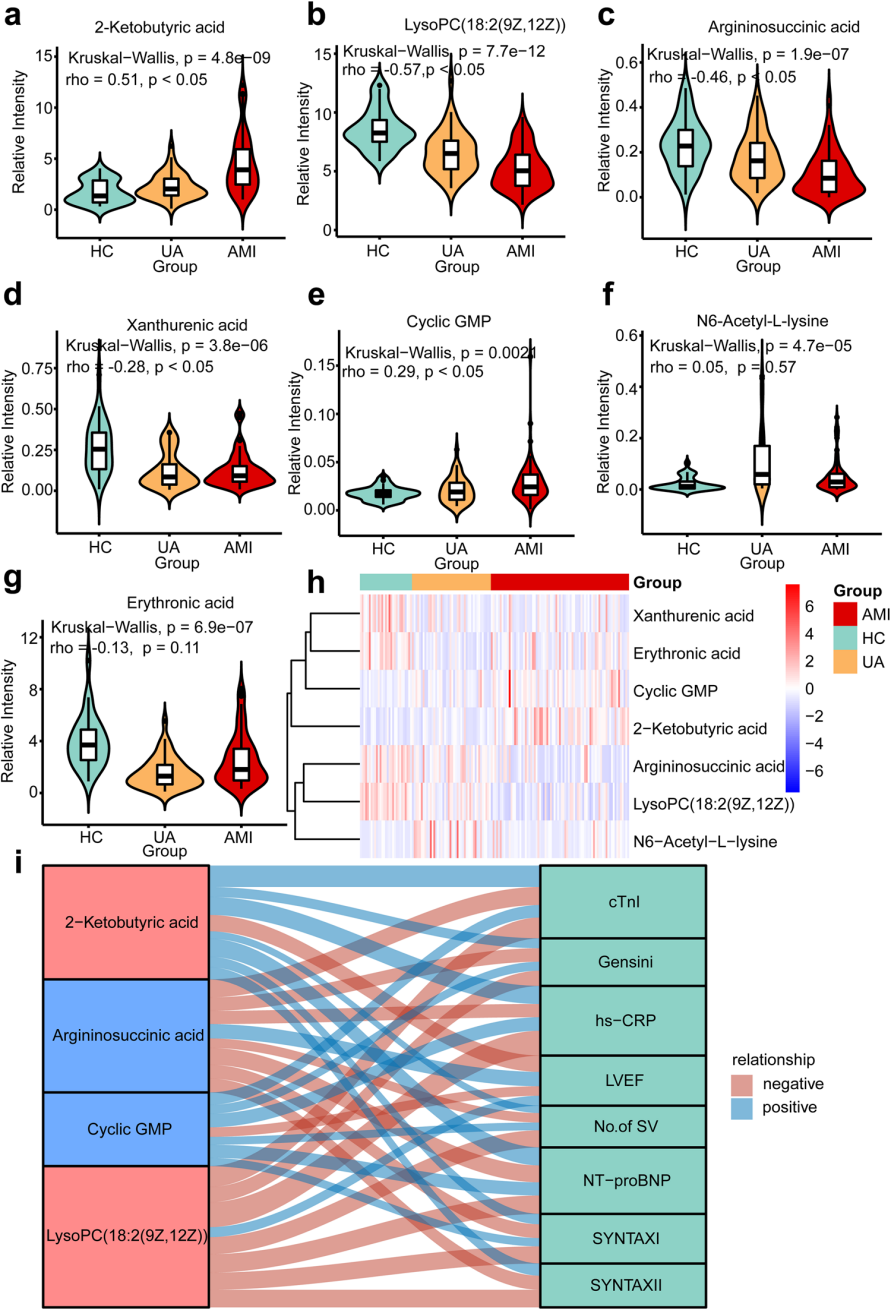
The potential metabolic biomarkers for the discrimination of the different ACS stages and correlations with ACS phenotypes. (**a**–**g**) The violin plot shows the distribution of 2-ketobutyric acid, LysoPC(18:2(9Z,12Z)), argininosuccinic acid, xanthurenic acid, cyclic GMP, N6-Acetyl-L-lysine, and erythronic acid in HC and different ACS stages. Spearman correlation analysis was used to reveal the relationship between metabolic signatures and the three progressive groups. The Kruskal–Wallis test was used to compare the three groups. (**h**) Heatmap and unsupervised cluster constructed using the seven metabolites. The blue color was of low abundance, and the red color was of high abundance. (**i**) According to Spearman correlation analysis, the alluvial plot shows the correlations between metabolic signatures and the ACS phenotype. The thickness of the connecting line indicates the magnitude of the correlation. The red stratums represent serum metabolites, and the blue stratums represent urine metabolites. Blue: positive correlation (*p* < 0.05); Red: negative correlation (*p* < 0.05).

To explore the correlation between the seven metabolic signatures and the ACS phenotype, we performed Spearman correlation analysis (Fig. 6). As shown in Fig. 6a,e, there was a progressive increase in 2-ketobutyric acid, and cyclic GMP as the disease progressed (rho = 0.51, *p* < 0.05; rho = 0.29, *p* < 0.05). LysoPC(18:2(9Z,12Z)), argininosuccinic acid, and xanthurenic acid showed a progressive decline as the disease progressed (rho = − 0.57, *p* < 0.05; rho = − 0.46, *p* < 0.05; rho = − 0.28, *p* < 0.05, Fig. 6b–d). The same pattern was not observed in N6-Acetyl-L-lysine and erythronic acid (rho = 0.05, *p* = 0.57; rho = − 0.13, *p* = 0.11, Fig. 6f,g). Unsupervised clustering analysis showed that 2-ketobutyric acid and cyclic GMP are more abundant in the AMI, while LysoPC(18:2(9Z,12Z)) and argininosuccinic acid are low-abundance in the AMI (Fig. 6h).

In order to further explore the relationship between the four metabolites and ACS phenotype, hs-CRP, cTnI, NT-proBNP, LVEF, SYNTAX score I, SYNTAX score II, Gensini score, and No. of SV were considered as indicators mirroring ACS severity. Spearman correlation analysis indicated that 2-ketobutyric acid was positively correlated with hs-CRP, cTnI, NT-proBNP, SYNTAX score I, SYNTAX score II, Gensini score, and the number of stenosed vessels (Fig. 6i, Supplemental Table S2). LysoPC(18:2(9Z,12Z)) and argininosuccinic acid were positively correlated with LVEF and negatively correlated with other indicators. Cyclic GMP was correlated with these indicators except for the SYNTAX score II.

### Integration of optimal diagnostic model

**Figure 7:**
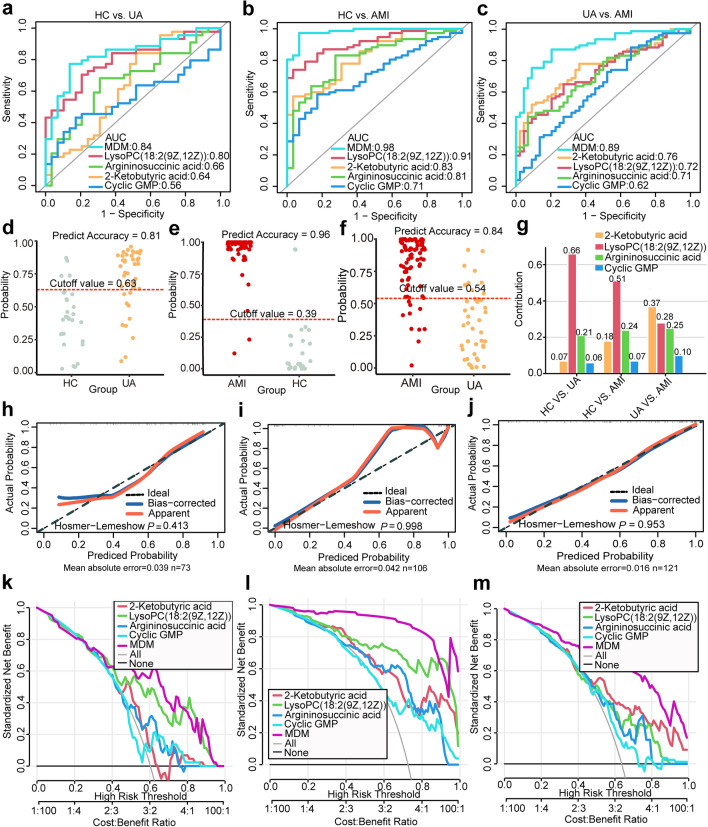
The diagnostic performance of the four potential metabolic biomarkers. (**a**–**c**) The diagnostic performance of 2-ketobutyric acid, LysoPC(18:2(9Z,12Z)), argininosuccinic acid, cyclic GMP, and metabolic diagnostic model (MDM) is shown via ROC curves for cross-comparisons among HC vs. UA, HC vs. AMI, and UA vs. AMI. (**d**–**f**) The optimal cut-off value for cross-comparisons among HC vs. UA, HC vs. AMI, and UA vs. AMI. The numbers above the red dashed line indicate the percentage of cases predicted as UA, or AMI. (**g**) Contribution of the four metabolites to MDM. (**h**–**j**) The calibration curves and Hosmer–Lemeshow test demonstrate good consistency between the predicted probability of MDM and the actual probability for HC vs.UA, HC vs. AMI, and UA vs. AMI in the internal validation with 1000 bootstrap repetitions. (**k**–**m**) Decision curve analysis for 2-ketobutyric acid, LysoPC(18:2(9Z,12Z)), argininosuccinic acid, cyclic GMP, and MDM in the comparisons of HC vs. UA, HC vs. AMI, and UA vs. AMI. The MDM was calculated by the combination of 2-ketobutyric acid, LysoPC(18:2(9Z,12Z)), argininosuccinic acid, cyclic GMP.

From the pathway analysis and the correlation with the ACS phenotype, we regarded the four metabolites, including 2-ketobutyric acid, LysoPC(18:2(9Z,12Z)), argininosuccinic acid, and cyclic GMP as candidate biomarkers of ACS and applied them to develop MDM. A ROC analysis was conducted to observe the diagnostic value of the four molecules. The area under the curve (AUC) of 2-ketobutyric acid, LysoPC(18:2(9Z,12Z)), argininosuccinic acid, and Cyclic GMP was 0.64, 0.80, 0.66, and 0.56, for distinguishing UA from HC (Fig. 7a). Encouragingly, the biomarkers panel MDM combined with the 4 metabolites displayed a more excellent effect on distinguishing UA from HC with an increased AUC of 0.84, and LysoPC(18:2(9Z,12Z)) contributed the most to UA diagnosis in the integrated model (Fig. 7a,g). The optimal cut-off value of MDM was 0.63 with a predictive accuracy of 81% (Fig. 7d). To recognize the AMI from the HC, the AUC of 2-ketobutyric acid, LysoPC(18:2(9Z,12Z)), argininosuccinic acid, and Cyclic GMP was 0.83, 0.91, 0.81, and 0.71 (Fig. 7b). The MDM was more excellent at distinguishing AMI (AUC = 0.98), and LysoPC(18:2(9Z,12Z)) contributed the most to AMI diagnosis (Fig. 7b,g). The optimal cut-off value was 0.39, with a predictive accuracy of 96% (Fig. 7e). The AUC of the above 4 molecules was 0.76, 0.72, 0.71, and 0.62 for identifying AMI from UA (Fig. 7c). The MDM exhibited significantly higher diagnostic performance (AUC = 0.89), and 2-ketobutyric acid contributed the most to distinguishing AMI from UA (Fig. 7c,g). The optimal cut-off value was 0.54, with a predictive accuracy of 84% (Fig. 7f).

To assess the consistency of the MDM, we performed the calibration curve by bootstrapping with 1000 resamplings in the internal validation. For the UA discrimination, the calibration curve presented excellent consistency between the actual and predicted probabilities of the MDM (mean absolute error = 0.039, Hosmer–Lemeshow *p* = 0.413, Fig. 7h). For the AMI discrimination, the simulated curve was consistent with the actual curve trajectory by performing 1000 resampling bootstraps (Hosmer–Lemeshow *p* = 0.998; Hosmer–Lemeshow *p* = 0.953; Fig. 7i,j). The calibration curves prove that the multiclass model MDM is not overfitted.

The DCA curves obtained for 2-ketobutyric acid, LysoPC(18:2(9Z,12Z)), argininosuccinic acid, cyclic GMP, and MDM for HC vs. UA, HC vs. AMI, and UA vs. AMI are presented in Fig. 7k–m. Compared with the single metabolite alone, the combination of four metabolites showed significantly higher net benefits, indicating that MDM has a potential clinical application value.

### Validation of the integrated model

We performed the bootstrapping with 1000 resamplings in the internal validation. The C-index with 1000 resampling was 0.83 (95% CI 0.73–0.91), 0.97 (95% CI 0.92–0.99), and 0.89 (95% CI 0.83–0.94) for HC vs. UA, HC vs. AMI, and UA vs. AMI (Supplemental Fig. S7a–c). Then we compared the single metabolite with the MDM by using 1000 resampling bootstraps in the internal validation (Table 2). NRI shows the diagnostic performance is improved by the MDM.

**Table 2:** Comparisons between single metabolite and MDM in the internal validation with 1000 bootstrap repetitions.

Exposure	AUC	95% CI	Specificity	Sensitivity	Accuracy	NRI
HC vs. UA						
2-Ketobutyric acid	0.65	0.50–0.75	0.48	0.84	0.70	0.31
LysoPC(18:2(9Z,12Z))	0.79	0.68–0.89	0.79	0.70	0.74	0.14
Argininosuccinic acid	0.66	0.53–0.77	0.69	0.68	0.68	0.26
Cyclic GMP	0.56	0.43–0.69	0.83	0.43	0.59	0.38
MDM	0.83	0.73–0.91	0.86	0.77	0.81	
HC vs. AMI						
2-Ketobutyric acid	0.82	0.73–0.89	0.97	0.57	0.68	0.37
LysoPC(18:2(9Z,12Z))	0.91	0.83–0.95	0.97	0.74	0.80	0.20
Argininosuccinic acid	0.80	0.70–0.88	0.72	0.83	0.80	0.35
Cyclic GMP	0.71	0.62–0.80	0.83	0.58	0.65	0.49
MDM	0.97	0.92–0.99	0.93	0.97	0.96	
UA vs. AMI						
2-Ketobutyric acid	0.76	0.68–0.83	0.91	0.52	0.66	0.24
LysoPC(18:2(9Z,12Z))	0.72	0.62–0.81	0.73	0.65	0.68	0.29
Argininosuccinic acid	0.71	0.61–0.80	0.93	0.44	0.62	0.29
Cyclic GMP	0.63	0.52–0.73	0.34	0.88	0.69	0.44
MDM	0.89	0.83–0.94	0.80	0.87	0.84	

*NRI* net reclassification improvement, *MDM* metabolic diagnostic model, calculated by 2-ketobutyric acid, *LysoPC(18:2(9Z,12Z))* argininosuccinic acid, and cyclic GMP.

### The relationship between serum and urine metabolites

We employed the correlation network to reveal the relationship between the 15 serum and 13 urine discriminate metabolic signatures (Supplemental Fig. S8). Uracil and caprylic acid in urine exhibit significant centralization, and this means that more serum metabolites are associated with them. N6-Methyladenosine and Phenylalanyl-Tryptophan were the most centralized serum metabolites. Proline betaine in serum shows the strongest positive correlation with vinylacetylglycine in urine (rho = 0.71, *p* < 0.05, Supplemental Fig. S8, Supplemental Table S3).

In addition, we observed that 7 out of the 28 potential discriminate metabolic signatures were detected in both blood and urine (Supplemental Fig. S9). Specifically, proline betaine in serum was positively correlated with it in urine, and exhibited similar upward trends from the HC to the AMI (R = 0.56, *p* = 2.2e−16, Supplemental Fig. S9c,j). N4-Acetylcytidine showed opposite trends in blood and urine (R = − 0.18, *p* = 0.026, Supplemental Fig. S9f,m).

2-Ketobutyric acid was the only one detected both in serum and urine among the four diagnostic biomarkers. The Spearman correlation analysis revealed that 2-ketobutyric acid in serum was not correlated with it in urine, and there was no regular change trend in serum and urine (R = 0.079, *p* = 0.34, Supplemental Fig. S9a,h). However, we noticed that the four diagnostic biomarkers were associated with other metabolites. Such as 2-ketobutyric acid was negatively correlated with uracil and caprylic acid, while LysoPC(18:2(9Z,12Z)) showed a positive correlation (Supplemental Fig. S8, Supplemental Table S3). Argininosuccinic acid was negatively related to N6-Methyladenosine, and positively related to Phenylalanyl-Tryptophan. Cyclic GMP was negatively related to LysoPC(22:0) (rho = − 0.30, *p* = 2.53E−04, Supplemental Fig. S8, Supplemental Table S3).

## Discussion

We applied UHPLC/QE-MS in an untargeted approach to measure metabolites in paired serum and urine samples from 150 participants. Then we performed unbiased machine-learning variable reduction techniques and adjusted for confounders to generate a novel multiclassification model that can play an essential role in stratifying ACS patients. By integrating 2-ketobutyric acid, LysoPC(18:2(9Z,12Z)), argininosuccinic acid, and cyclic GMP, we established the multiclassification model MDM that approached a strong C-index, outperforming single metabolite alone. Furthermore, we discovered that the four metabolites changed dynamically as plaque burden increased. In addition, 2-ketobutyric acid is identified both in serum and urine, but they are not correlated. These results lend credence to the idea that the combination of serum and urine metabolites exhibits powerful efficiency in classifying subjects into HC, UA, or AMI, which may have the clinical effect of improving risk stratification relatively noninvasively.

The multinomial classifiers named adaptive LASSO multinomial regression and RF for feature selection employed in our research are differentiated from previous research^36–38^. The aim of this study is to establish a multiclassification metabolic model to classify subjects into HC, UA, or AMI. Obviously, this is a multiclassification problem (N > 2) rather than a simple binary classification problem. Multiclass metabolic models are scarce in the field of CAD. As the complexity of determining accurate class decision boundaries increases, it is more difficult to obtain stable and reliable classification models for multiclassification^21, 22^. Therefore, compared with binary classification, machine learning algorithms suitable for multiclassification are greatly reduced^23^. A report claimed that fewer than fourteen ML methods can be applied to multiclass metabolomics^23^. The premise of applying machine learning algorithms in the past multiclassification metabolic research of CAD is to convert the multiclassification into a dichotomous problem through cross comparison and traditional hypothesis testing, such as the Student's *t*-test^16, 24^. These studies used RF, binominal LASSO, and support vector machine (SVM) to construct binary classification so as to overlook the establishment of multiclassification models^16, 24^. Traditional hypothesis testing will narrow the prior range of differential metabolites, and multiple pairwise comparisons will increase the risk of type I error^39^. In view of this, this study does not use traditional hypothesis testing and multiple pairwise comparisons in the modeling process. Adaptive LASSO is an upgraded version of LASSO that can overcome the shortcomings of LASSO, as it obtains initial weights using ordinary least squares estimation, which results in higher penalties for zero coefficients and lower penalties for nonzero coefficients^36, 40^. We ameliorated adaptive LASSO into multinomial adaptive LASSO, and combined it with RF to build a multiclass metabolic model to classify HC, UA, and AMI. We plugged all identified metabolites into the multinomial classifiers for feature selection, which was conducive to making comprehensive use of metabolites and eliminating type I error. Finally, 2-ketobutyric acid, LysoPC(18:2(9Z,12Z)), argininosuccinic acid, and cyclic GMP stood out as representatives of ACS after controlling confounders and the interaction test, potentially mirroring ACS severity.

The selected metabolic small molecules based on different machine learning algorithms require reproducibility. Previous studies have found that one of the reasons for the low reproducibility of biomarkers is due to the inappropriate methods of identifying biomarkers^41^. The reproducibility of biomarkers in different populations, subgroups, or subsets is the fundamental criterion for measuring the performance of machine learning algorithms used^42, 43^. Based on the reproducibility criteria, this study obtained candidate diagnostic metabolic small molecules by taking intersections based on multinomial adaptive LASSO regression and RF and then conducting confounding factor evaluation and interaction testing. In a real-world setting, it is almost impossible to completely remove the influence of confounding factors on host metabolism. Our study identified independent metabolites that are not modified by age, sex, BMI, smoking status, history of hypertension or diabetes, or levels of TG, TC, HDL-C, and LDL-C to the maximum extent. Whether these metabolites are affected by other potential confounders was not evaluated in this study. The representativeness of the study population is another important factor affecting reproducibility. The importance of sample size for the generalizability of results to a broader population is widely acknowledged. The sample size of this study meets the sample size requirement for identifying differences in metabolites between groups. This indicates that the study population is representative at the sample size level. In addition, the baseline characteristics of the population in this study are consistent with the epidemiological characteristics of ACS, which proves that the study population is representative to some extent^44^. Meanwhile, model overfitting is one of the difficulties in applying machine learning to small datasets^45^. Although this multiclassification model did not undergo external dataset validation for reproducibility, internal validation, including cross-validation and 1,000 resampling bootstraps, demonstrated that it has considerable reproducibility and there is no overfitting of the model.

**Figure 8:**
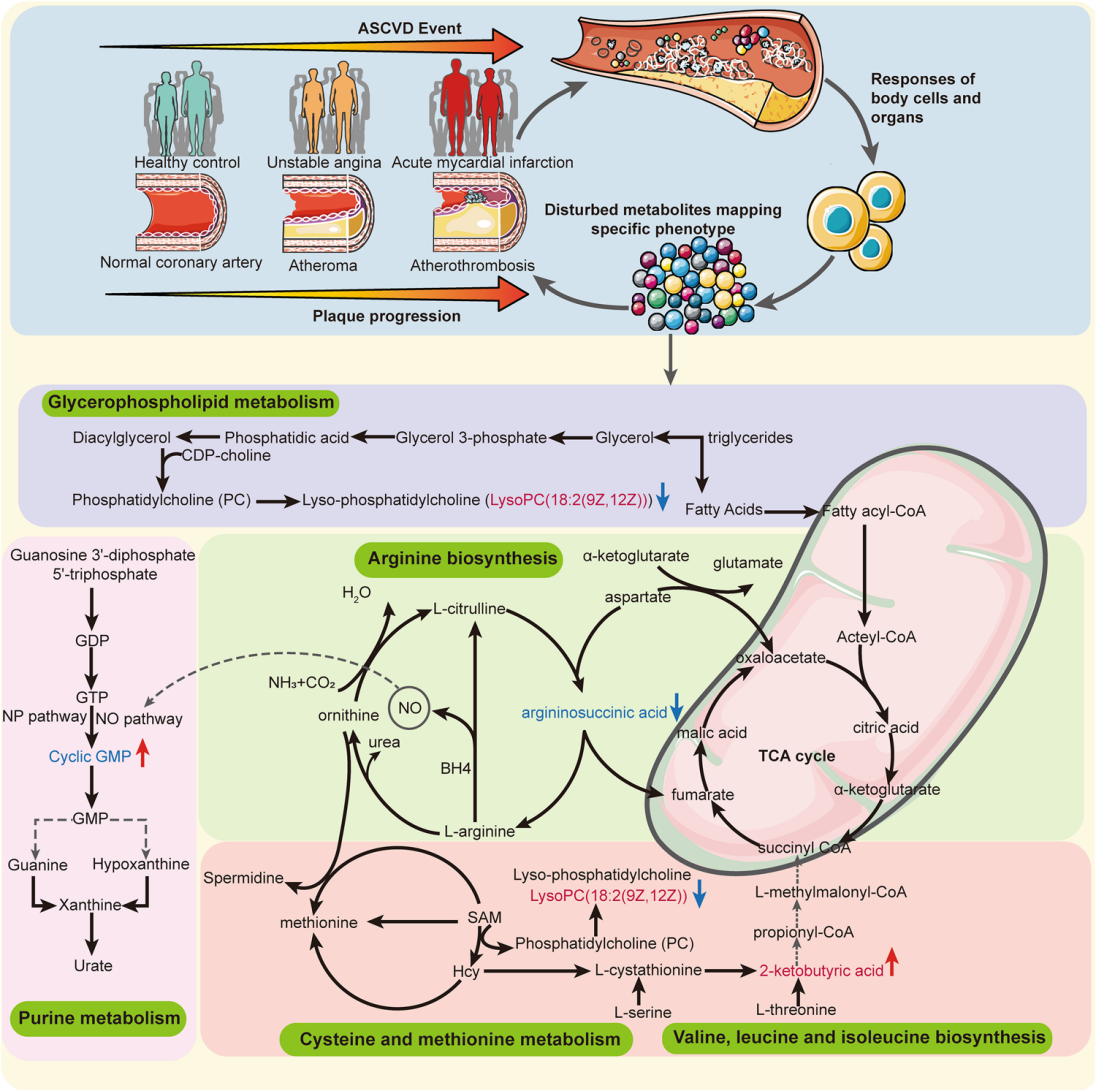
Disturbed metabolic pathways implicated in ACS pathogenesis. Five metabolic pathways relating to MDM during the progression from HC to UA and then to AMI are altered: glycerophospholipid metabolism, cysteine and methionine metabolism, valine, leucine and isoleucine biosynthesis, arginine biosynthesis, purine metabolism. Red font indicates the two-serum potential diagnostic metabolites, and the blue indicates the two-urine potential diagnostic metabolites. *NP pathway* natriuretic peptide pathway, *NO pathway* nitric oxide pathway.

We mapped biochemical metabolic pathways based on the four metabolic signatures (Fig. 8). Glycerophospholipid metabolism in serum and arginine biosynthesis in urine were the two most significantly altered pathways in the transition of HC → UA → AMI. This demonstrated that a significant disorder emerged in glycerophospholipid metabolism and arginine biosynthesis as ACS progressed. Our findings were supported by previous research^15, 46, 47^. These previous studies showed that glycerophospholipid metabolism was found to be the most significantly altered metabolic pathway in all paired comparisons, such as UA versus SA, AMI versus UA^15, 48^. Consistent with our results, glycerophospholipids in SA and AMI patients were significantly lower than those in healthy individuals^49^. Glycerophospholipid metabolism is closely related to the inflammatory response of CAD, and glycerophospholipids may act as potential inflammatory mediators^47, 50^. The arginine biosynthesis pathway represents the source of nitric oxide (NO) production^51^. Reduced arginine uptake can cause a decrease in NO bioavailability^51^. NO exerts anti atherosclerotic cardioprotective effects by vasodilation, inhibiting platelet aggregation, and adhesion, inhibiting inflammation produced by leukocyte adhesion to blood vessels, and inhibiting vascular smooth muscle proliferation^52^. Previous studies have confirmed that the arginine metabolic pathway in CAD is impaired, which supports our findings^15, 47^. Some scholars claim that exogenous arginine supplementation can restore NO bioavailability, increase coronary blood flow, dilate blood vessels, and relieve angina pectoris in patients with CAD, while others hold negative results^51, 53^. Therefore, the benefit of arginine for CAD is controversial.

As Fig. 8 depicts, the tricarboxylic acid (TCA) cycle distributed in the mitochondria is like a critical hub linking the metabolic disturbances of 2-ketobutyric acid and arginosuccinic acid. Compared with the other three metabolites, 2-ketobutyric acid contributed the most to the MDM for differentiating AMI from UA, and was positively correlated with plaque burden and myocardial injury indicators. This implies that 2-ketobutyric acid is of the highest value in the diagnosis of UA and AMI. 2-Ketobutyric acid may be an agonist of atherosclerosis and myocardial injury or may be a consequence. As a kind of α-keto acid, 2-ketobutyric acid can be converted into succinyl CoA and then enter the TCA cycle ^54^. A magnitude of studies identified the impaired TCA cycle in myocardial ischaemia, which is similar to our results^5, 55, 56^. Previous studies reported a decrease in intermediate metabolites of the TCA cycle during AMI, such as fumaric acid, succinate, and oxaloacetic acid^57, 58^. However, some scholars are opposed to this. They believe that succinate is elevated during myocardial ischaemia and drives reactive oxygen species (ROS) to cause ischaemia–reperfusion injury^56, 59, 60^. In line with the previous study, our result demonstrated that the levels of 2-ketobutyric acid were upregulated in ACS^13^. Researchers speculated that accumulations of 2-ketobutyric acid, the TCA cycle intermediate, signified perturbations of oxidative phosphorylation^61^. Activating 2-ketobutyric acid production was sufficient to promote glucose oxidation and mitochondrial respiration^62^. The accumulation of 2-ketobutyric acid in ACS, especially in AMI, may be due to the feedback increases caused by the impaired TCA cycle activity.

In this work, higher levels of argininosuccinic acid were associated with a lower risk of ACS. We showed that argininosuccinic acid was negatively correlated with ACS phenotype indices. This indicates that the deficiency of argininosuccinic acid might have a deleterious effect on ACS. Thus, our findings suggest that argininosuccinic acid is beneficial for ACS and it may contribute to the etiology of ACS as part of the cause or a consequence. The specific causality needs further verification. Arginosuccinic acid can be synthesized from citrulline and is used as a precursor of arginine in arginine biosynthesis. This study clarifies that arginosuccinic acid is involved in the progression of HC → UA → AMI via dysregulated arginine biosynthesis. The previous study supported our view by showing that a higher arginine/asymmetric dimethylarginine ratio was associated with lower CVD incidence^63^. It was reported that a supplement of L-citrulline, a precursor of argininosuccinic acid, showed benefits for cardiovascular and metabolic health outcomes^64^. This report supports our findings. As a precursor of fumarate, the downregulation of argininosuccinic acid in the ACS might indirectly affect fumarate production and further signify abnormal activities of the TCA cycle.

LysoPC(18:2(9Z,12Z)), an unsaturated LPC (lysophosphatidylcholine), participates in the progression of HC → UA → AMI via the dysregulated glycerophospholipid metabolism, which is supported by the previous research^46^. We find that LysoPC(18:2(9Z,12Z)) contributes the greatest diagnostic value in distinguishing whether an individual suffers from ACS or not with the maximum contribution ratio. Interestingly, we observed that LysoPC(18:2(9Z,12Z)) was in low abundance in AMI and was negatively associated with atherosclerosis plaque burden and hs-CRP. This analysis provides insight into the anti-atherosclerotic and anti-inflammatory properties of LysoPC(18:2(9Z,12Z)) in the setting of ACS^65^. Consistent with our findings, previous studies showed that LysoPC(18:2) was downregulated in the ACS or in the AMI^15, 66^. Large-scale cohort studies suggested a negative association between LysoPC(18:2) and MACE risk or hs-CRP, which supported our findings^46, 67^. A previous study demonstrated that LPC can bind C-reactive protein (CRP), thereby inhibiting its pro-atherogenic effect on macrophages and delaying the progression of atherosclerosis^68^. These discoveries demonstrate that LysoPC(18:2) could stabilize atherosclerotic plaque. This contradicts LPC could significantly induce the uptake of oxLDL by macrophages, thereby transforming into foam cells and aggravate the deterioration of atherosclerotic plaques^69^. The following reasons can explain: LPC may play an antiatherosclerosis role by inhibiting cholesterol biosynthesis and reducing cellular cholesterol accumulation in liver cells^70^ and macrophages^71^. Another explanation for this discrepancy result is attributed to that the acyl chain length and saturation of different LPC species affect their activity and function^72, 73^. Saturated LPC, such as LPC (16:0), can promote the release of inflammatory cytokines^74^. Unsaturated LPC, such as LPC (20:4), can inhibit the production of inflammatory mediators and exert anti-inflammatory effects, which supports our findings^74^. Recent evidence elucidates that the more the number of double bonds of phosphatidylcholine, the lower the risk of death from CAD^46^. This supports the view of this study that polyunsaturated LPC, such as LysoPC(18:2(9Z,12Z)), have beneficial effects on ACS.

Another intriguing discovery is that higher urinary cyclic GMP (cGMP) levels were associated with an increased risk of incident ACS and positively related to atherosclerosis plaque burden. Consistent with our result, a case-cohort analysis nested study with 875 participants showed that higher levels of cGMP were independent risk factors for heart failure, cardiovascular events, and coronary heart disease after 9.9 years of follow-up^75^. Activating the NO/cGMP/cGMP-dependent protein kinase type I (cGKI)-signaling pathway can facilitate thrombus dissolution^76^. Elevating cGMP provides protection against ischaemia and reperfusion injury by increasing Ca^2+^-activated K^+^ channels of the BK-type activity (BK)^77^. These protective effects of cGMP seem to contradict the positive association between urinary cGMP and CVD risk. Some reasons can explain this relationship. Direct activation of the respective guanylate cyclase by nitric oxide (NO) or natriuretic peptide (NP)-triggered pathways can upregulate cGMP^78^. The increase of cGMP in urine was largely triggered by the NP pathway rather than NO pools^79^. We observed that cGMP was positively correlated with NT-proBNP, a natural ligand for membrane-bound guanylate cyclase receptors to stimulate the synthesis of cGMP. This result supports the idea that urinary upregulated cGMP in ACS is more likely to originate from the NP pathway. Although the cardioprotective effects of cGMP, cGMP levels are upregulated in ACS pathological conditions, like NT-proBNP. The elevated urinary cGMP reflects inadequate compensatory mechanisms of ACS. In addition, by analyzing the relationship between serum and urine metabolites, we found that cGMP was detected only in urine and not in serum. This is due to the fact that cGMP produced by the NO pathway is intracellular compartmentalized and difficult to measure in blood, while cGMP produced by the NP pathway resides at the membrane and is secreted to the extracellular^80^. This is another explanation for our detection of elevated urine cGMP levels in ACS. This paradox highlights the complexity of the relationship between cGMP and ACS.

One of the strengths of the study is that the detailed contribution proportions of the four metabolites to MDM are helpful for clinical decision-making. These metabolites have the potential to be made into reagents to monitor the occurrence of ACS. Their clinical application value is reflected in that when an individual's 2-ketobutyric acid is the most significantly upregulated among the four signatures, they are at high risk of AMI rather than UA, and when LysoPC(18:2(9Z,12Z)) is most significantly downregulated, they are more inclined to UA or AMI diagnosis rather than healthy status. Our results fill a gap in the establishment of a multiclassification metabolomics model with multinomial ML approaches, especially multinomial adaptive LASSO regression. Furthermore, the MDM is a robust independent biomarker panel of ACS and atherosclerosis plaque burden, as the spurious effects of some confounding factors were dismissed.

Further investigations are essential. This is a cross-sectional study, and only the metabolites at a certain moment were observed. Long-term dynamic metabolite changes or metabolic flux using tracer technology is needed to determine whether there is a causal relationship between the four metabolic signatures and ACS. Besides, a large number of metabolites used to prepare diagnostic test reagents do not have reference material. At present, one of the biggest challenges and limitations of metabolome disease diagnostics is the generalizability of target metabolites, which can be affected by the diversity of the population, genetics, environment, and various potential confounders^7^. This study serves as an exploratory analysis, a larger-scale cohort study for external validation in real-world medicine is essential to proving the generalizability and reproducibility of our findings.

## Conclusion

In conclusion, glycerophospholipid metabolism and arginine biosynthesis act on predominant roles as ACS and atherosclerosis plaque progression. This study provides a new metabolic multiclassification model and multinomial machine learning algorithm for ACS. The MDM integrated by 2-ketobutyric acid, LysoPC(18:2(9Z,12Z)), argininosuccinic acid, and cyclic GMP have emerged as robust, independent hallmarks of ACS and atherosclerosis plaque. Our finding provides new insights for revealing novel potential etiologies for ACS.

### Supplementary Information

Supplementary Figures.

Supplementary Tables.

## Data Availability

The original data presented in the study are included in the article/Supplementary Materials.

## Abbreviations

HC: Healthy controls
UA: Unstable angina
AMI: Acute myocardial ischaemia
BMI: Body mass index
WBC: White blood cell count
NEUT: Neutrophil count
LYM: Lymphocyte
RBC: Red blood cell count
HGB: Hemoglobin
PLT: Platelet count
BUN: Blood urea nitrogen
Scr: Serum creatinine
UA: Uric acid
eGFR: Estimated glomerular filtration rate
Ccr: Endogenous creatinine clearance rate
Glu: Glucose
AST: Aspartate aminotransferase
ALT: Alanine transaminase
TG: Triglyceride
TC: Total cholesterol
HDL-C: High-density lipoprotein cholesterol
LDL-C: Low-density lipoprotein cholesterol
HCY: Homocysteine
hs-CRP: Hypersensitive C-reactive protein
cTnI: Cardiac troponin I
NT-proBNP: N-terminal pro-brain natriuretic peptide
LVEF: Left ventricular ejection fraction
CI: Confidence interval
OR per SD: Per-standard deviation odds ratio
ROC: Receiver operating characteristics curve
AUC: Area under the curve
No. of SV: Number of stenosed vessel
NRI: Net reclassification improvement
MDM: Metabolic diagnostic model
