# Supplementary material for: Multinomial machine learning identifies independent biomarkers by integrated metabolic analysis of acute coronary syndrome
Source: Sci Rep. 2023 Nov 23;13:20535. doi: 10.1038/s41598-023-47783-5 (PMC10667512; doi:10.1038/s41598-023-47783-5)
Supplement: Supplementary file 1 — Supplementary Figures. [file 41598_2023_47783_MOESM1_ESM.pdf]

Supplementary Figures

Multinomial machine learning identifies independent biomarkers by integrated metabolic analysis of acute coronary syndrome

Meijiao Fu [1, 2]; Ruhua He [1]; Zhihan Zhang [3]; Fuqing Ma [4]; Libo Shen [5]; Yu Zhang [2]; Mingyu Duan [2]; Yameng Zhang [6]; Yifan Wang [7]; Li Zhu [7*]; Jun He [1*]

Author affiliations:

[1] Department of Cardiology, General Hospital of Ningxia Medical University, Yinchuan, Ningxia 750004, China;

[2] Ningxia Medical University, Yinchuan, Ningxia 750004, China;

[3] Department of Cardiology, Hanzhong Central Hospital, Hanzhong, Shanxi 723200, China;

[4] Department of Cardiology, The Fifth People's Hospital of Ningxia, Shizuishan, Ningxia 753000, China;

[5] Center for Cardiovascular Diseases, People's Hospital of Ningxia Hui Autonomous Region, Yinchuan, Ningxia 750002, China;

[6] Department of Cardiology, The Second Affiliated Hospital of Henan University of Science and Technology, Luoyang, Henan 471000;

[7] Department of Radiology, General Hospital of Ningxia Medical University, Yinchuan, Ningxia 750004, China;

***Corresponding author: Li Zhu and Jun He**

Li Zhu

Department of Radiology, General Hospital of Ningxia Medical University, Yinchuan, Ningxia 750004, China;

Email: zhuli72@163.com

Jun He

Department of Cardiology, General Hospital of Ningxia Medical University, Yinchuan, Ningxia 750004, China;

Email: junhe@nyfy.com.cn

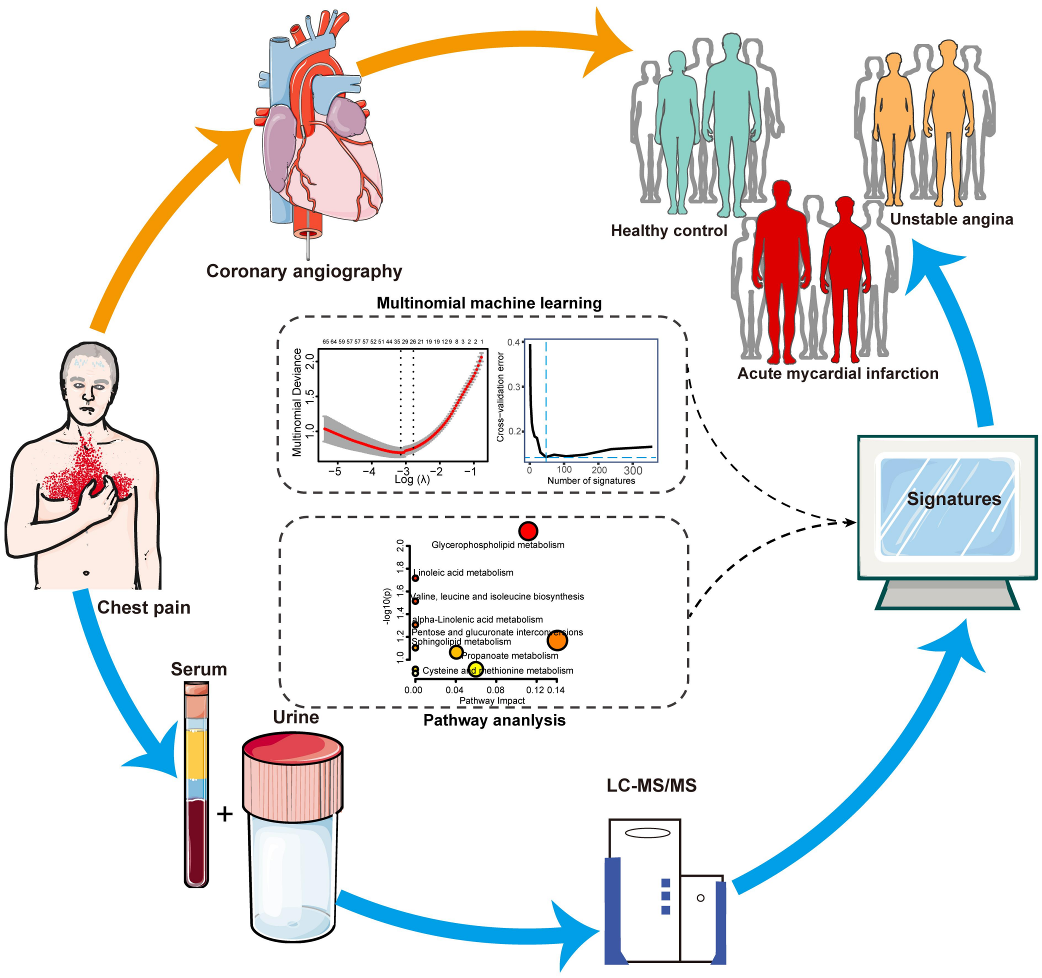

Supplemental Figure S1 Graphical Abstract

MATERIALS AND METHODS

Sample size calculation

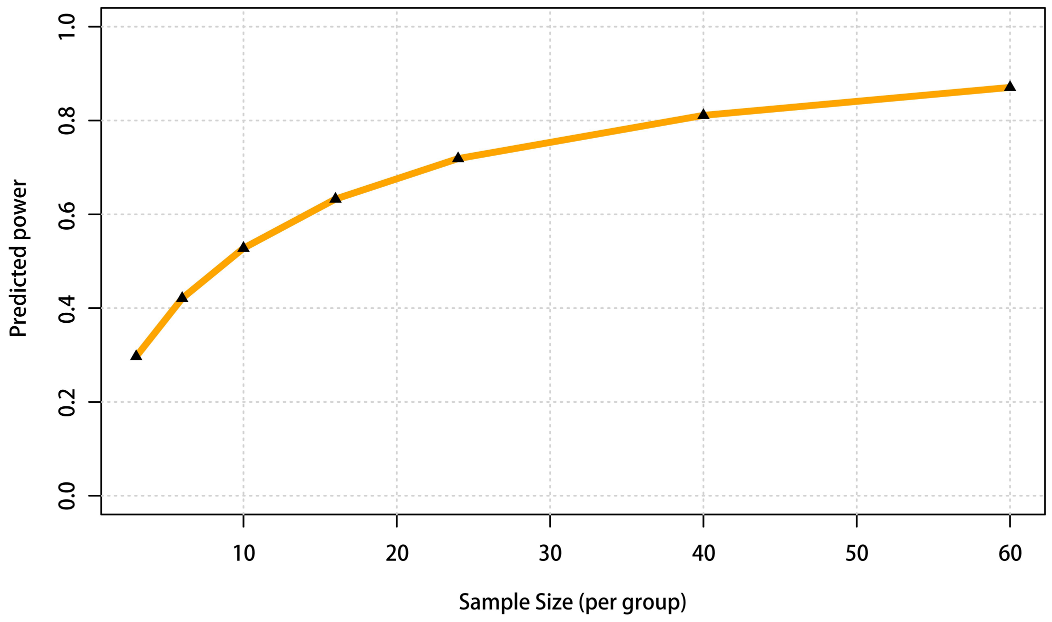

Supplemental Figure S2 Power analysis

The power analysis suggests that we will have over 80% power to detect discriminating metabolites using approximately 40 subjects per group. And we will have over 75% power to detect discriminating metabolites using approximately 30 subjects per group.

We performed power analysis using MetaboAnalyst software v5.0. A false discovery rate (FDR) of 0.1 was chosen as the significance criterion. We estimated the sample size using HC (healthy control) and AMI (acute myocardial infarction). Power in our samples reaches an acceptable level (0.8) at a sample size of approximately 120 (per group ≈ 40) (**Supplementary Figure S2**). If power is set at 0.75, the total sample size is approximately 90 (per group ≈ 30) (**Supplementary Figure S2**). We actually

recruited 150 subjects, which meet sample size standards. The study population is representative at the sample size level".

RESULTS

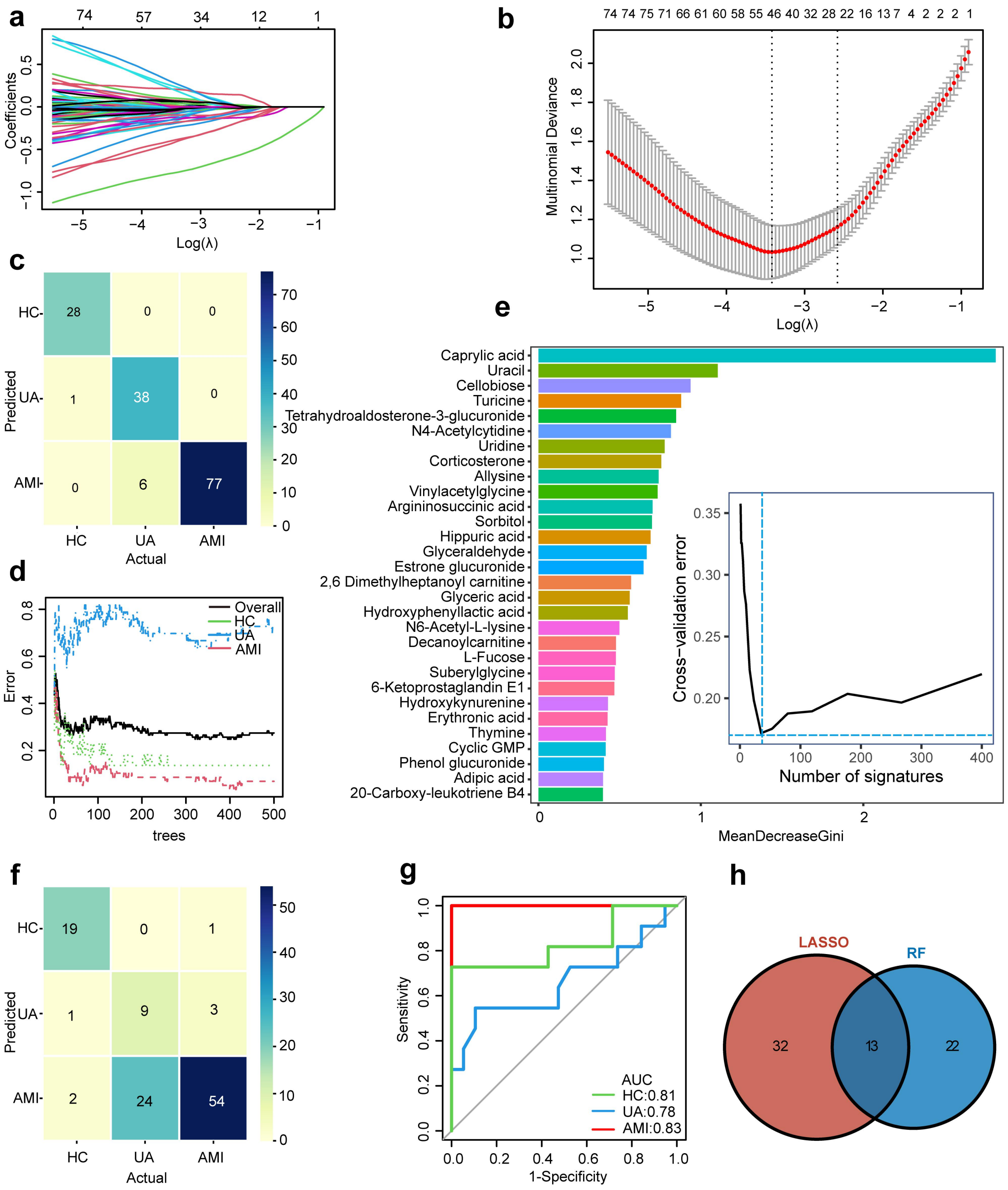

Supplemental Figure S3 The 13 candidate urine metabolic biomarkers selected by adaptive LASSO multinomial regression and random forest algorithms. (a) Plots for adaptive LASSO multinomial regression coefficients over different values of

the penalty parameter λ. **(b)** Cross-validation plots for the penalty parameter λ. The 45 candidate metabolic signatures were chosen by adaptive LASSO multinomial regression (λ_{min} =0.03). **(c)** The confusion matrix of the internal cross-validation set shows that 28 HC, 38 UA, and 77 AMI were correctly classified by the adaptive LASSO multinomial algorithm. **(d)** The correlation plots between the number of RF trees and model error show the error stabilized when using 500 trees. **(e)** The top 30 discriminant metabolic signatures are ranked in descending order of importance to the accuracy of the RF classifier for HC, UA, and AMI. The insert represents the minimum cross-validation error obtained when using 35 signatures by performing 10-fold cross-validation with five repeats. **(f)** The confusion matrix of the training set (n =113) based RF algorithms shows 82 subjects are correctly classified. **(g)** The multinomial ROC curves based on RF algorithms are used to distinguish HC, UA, and AMI in the internal test set (n =37)**.** **(h)** The Venn diagram shows the 13 overlapped biomarkers selected by adaptive LASSO multinomial regression and RF algorithms

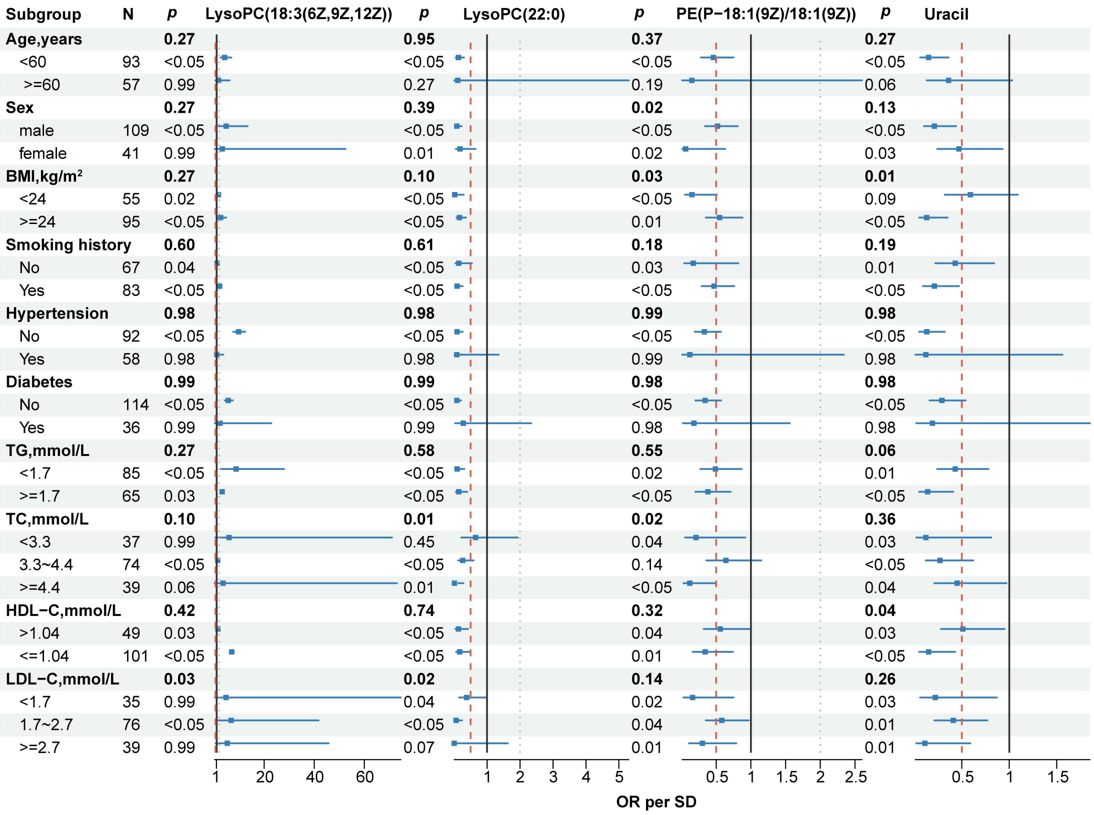

Supplemental Figure S4 Subgroup analysis of LysoPC(18:3(6Z,9Z,12Z)), LysoPC(22:0), PE(P-18:1(9Z)/18:1(9Z)), and uracil

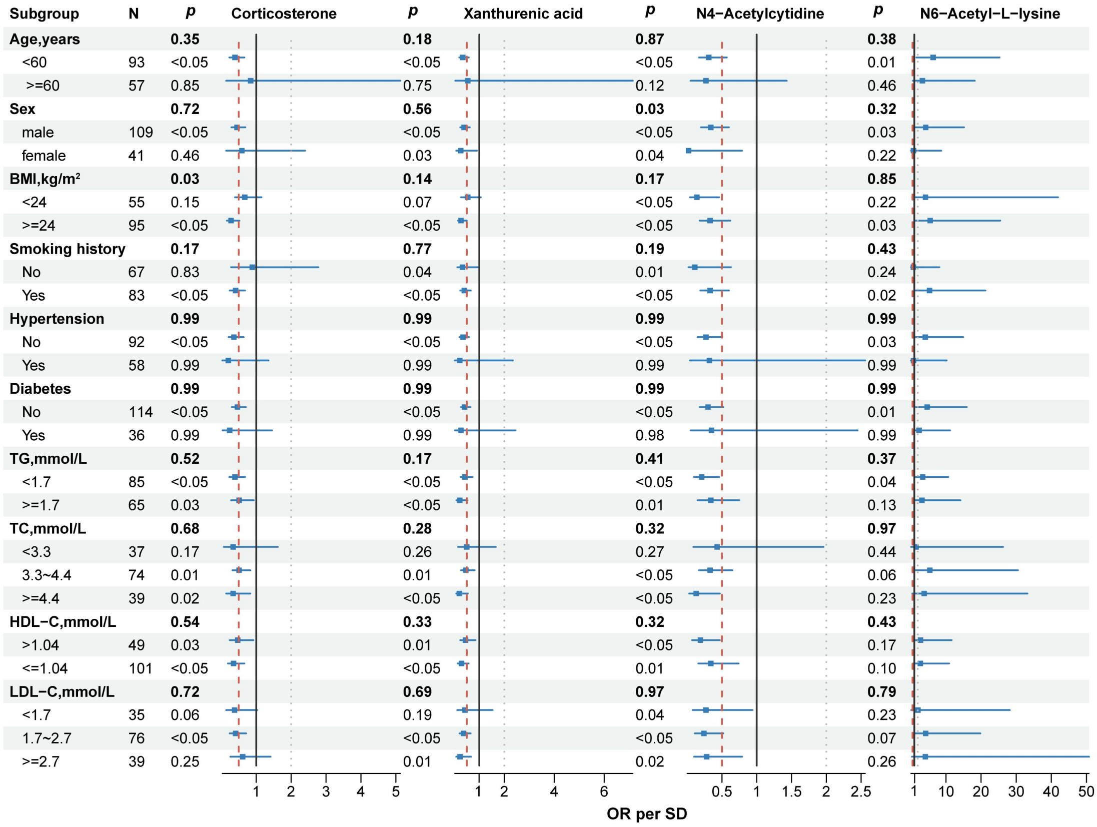

Supplemental Figure S5 Subgroup analysis of corticosterone, xanthurenic acid, N4-Acetylcytidine, and N6-Acetyl-L-lysine

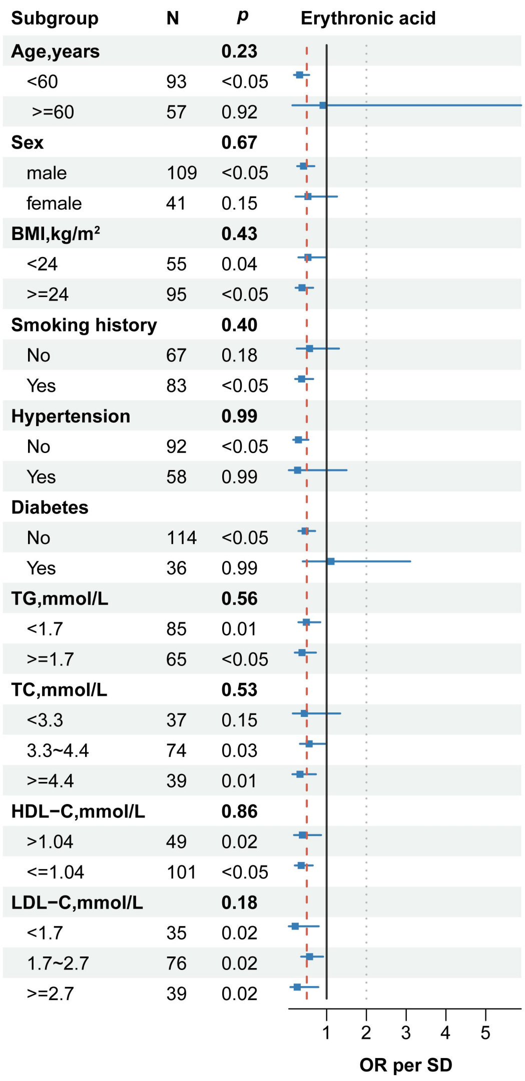

Supplemental Figure S6 Subgroup analysis of erythronic acid

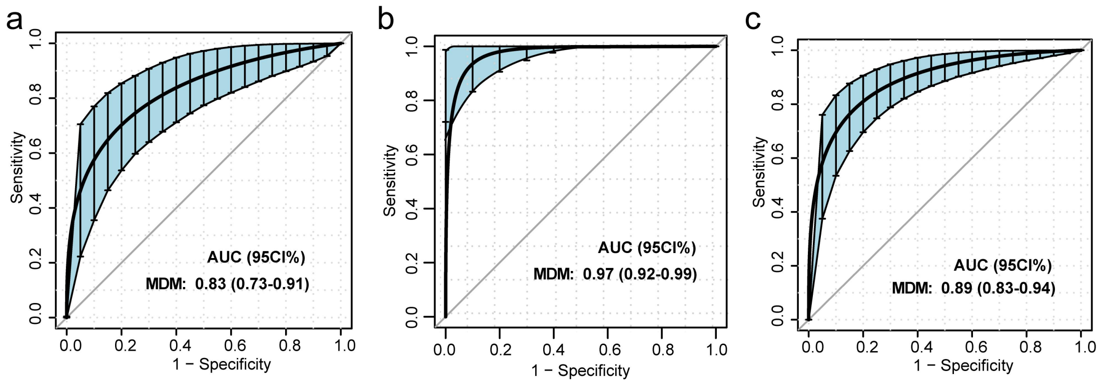

Supplemental Figure S7 The internal validation of MDM. (a-c) The C-index demonstrated excellent discriminatory power of MDM in the internal validation set:

HC vs. UA, HC vs. AMI, and UA vs. AMI. The blue shadow means the 95% CI of the C-index, calculated by 1000 resampling bootstraps.

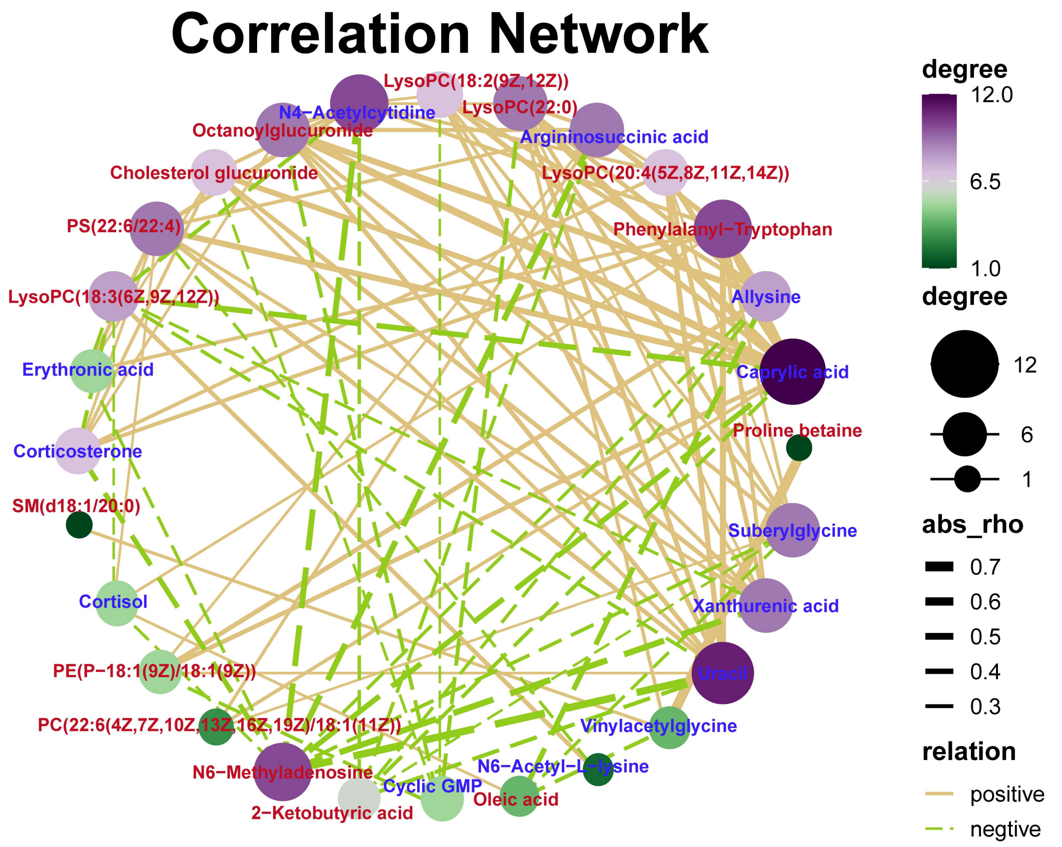

Supplemental Figure S8 The relationship between serum and urine metabolites. The network calculated by the Spearman correlation analysis displayed the relationships between the 15 serum and 13 urine metabolic signatures. The size and color of the dots indicate the degree of the metabolites' centralization. The larger and darker the dot, the more metabolites are associated with it. Line thickness indicates the |rho|. The solid yellow lines indicate positive correlations, and the dashed green lines indicate negative correlations. The color of metabolites in red represents serum metabolites, and blue represents urine metabolites. The network shows |rho| > 0.2, $p < 0.05$.

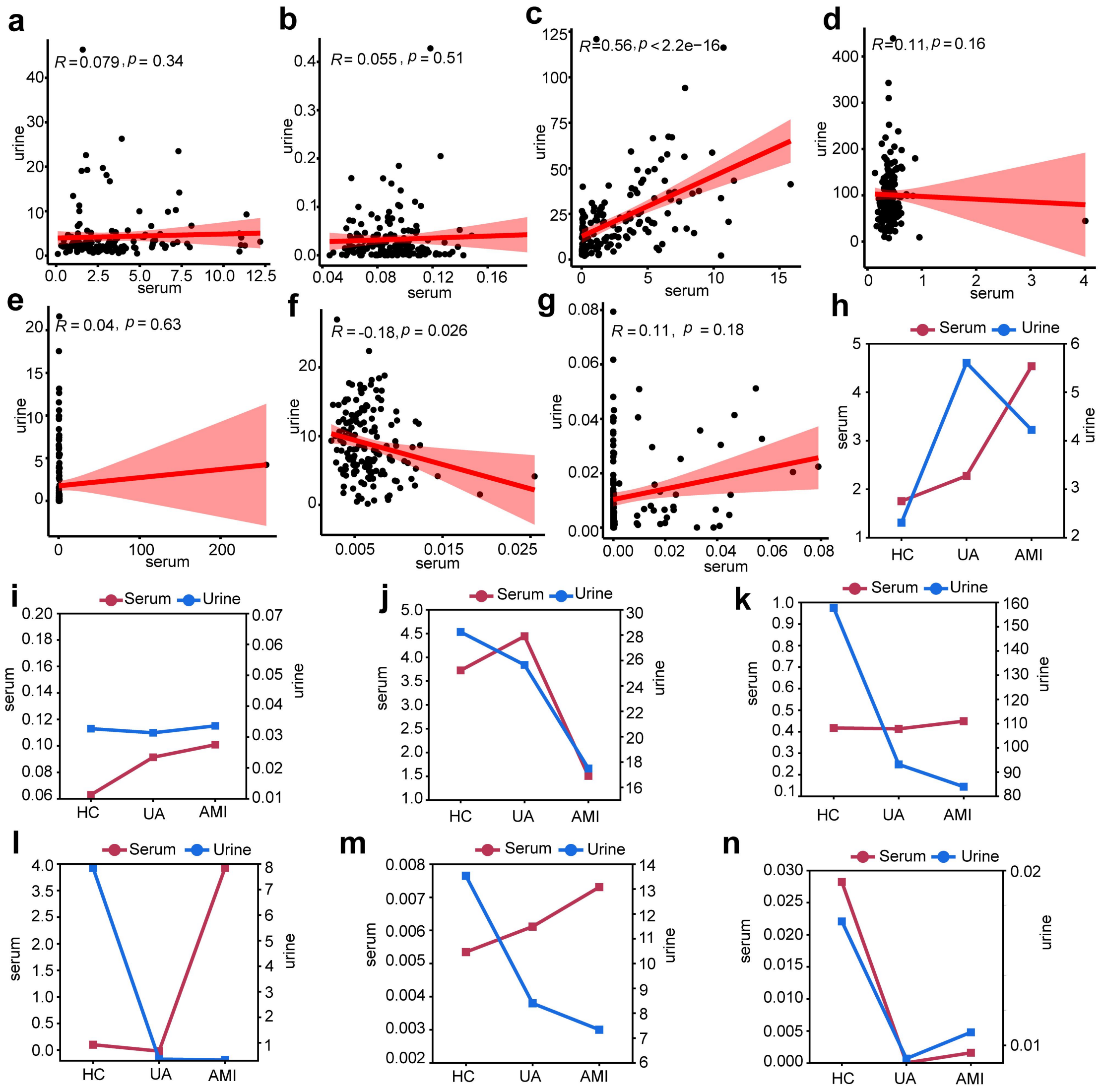

Supplemental Figure S9 The relationship between serum and urine metabolites.

(a-g) Correlation analysis of 2-ketobutyric acid, N6-Methyladenosine, proline betaine, uracil, caprylic acid, N4-Acetylcytidine, and cortisol in serum and urine. The 7 metabolites were detected both in serum and urine. The proline betaine level in serum is positively related to that in urine, and N4-Acetylcytidine level shows the reverse relation. **(h-n)** The trend of 2-ketobutyric acid, N6-Methyladenosine, proline betaine, uracil, caprylic acid, N4-Acetylcytidine, and cortisol in blood and urine during the three groups.
