# Supplementary material for: Multinomial machine learning identifies independent biomarkers by integrated metabolic analysis of acute coronary syndrome
Source: Sci Rep. 2023 Nov 23;13:20535. doi: 10.1038/s41598-023-47783-5 (PMC10667512; doi:10.1038/s41598-023-47783-5)
Supplement: Supplementary file 2 — Supplementary Tables. [file 41598_2023_47783_MOESM2_ESM.pdf]

Supplementary Tables

Multinomial machine learning identifies independent biomarkers by integrated metabolic analysis of acute coronary syndrome

Meijiao Fu [1, 2]; Ruhua He [1]; Zhihan Zhang [3]; Fuqing Ma [4]; Libo Shen [5]; Yu Zhang [2]; Mingyu Duan [2]; Yameng Zhang [6]; Yifan Wang [7]; Li Zhu [7*]; Jun He [1*]

Author affiliations:

[1] Department of Cardiology, General Hospital of Ningxia Medical University, Yinchuan, Ningxia 750004, China;

[2] Ningxia Medical University, Yinchuan, Ningxia 750004, China;

[3] Department of Cardiology, Hanzhong Central Hospital, Hanzhong, Shanxi 723200, China;

[4] Department of Cardiology, The Fifth People's Hospital of Ningxia, Shizuishan, Ningxia 753000, China;

[5] Center for Cardiovascular Diseases, People's Hospital of Ningxia Hui Autonomous Region, Yinchuan, Ningxia 750002, China;

[6] Department of Cardiology, The Second Affiliated Hospital of Henan University of Science and Technology, Luoyang, Henan 471000;

[7] Department of Radiology, General Hospital of Ningxia Medical University, Yinchuan, Ningxia 750004, China;

***Corresponding author: Li Zhu and Jun He**

Li Zhu

Department of Radiology, General Hospital of Ningxia Medical University, Yinchuan, Ningxia 750004, China;

Email: zhuli72@163.com

Jun He

Department of Cardiology, General Hospital of Ningxia Medical University, Yinchuan, Ningxia 750004, China;

Email: junhe@nyfy.com.cn

Supplemental Table S1 Univariable and multivariable analyses of the associations between metabolites and ACS risk

	Crude model		Adjust I		Adjust II	
	**OR (95%CI)**	***p value***	**OR (95%CI)**	***p value***	**OR (95%CI)**	***p value***
**Serum metabolites**						
Proline betaine	0.72 (0.49-1.04)	0.08	0.65 (0.42-1.01)	0.05	0.55 (0.26-1.15)	0.11
SM(d18:1/20:0)	1.05 (0.70-1.59)	0.81	0.97 (0.61-1.54)	0.90	0.66 (0.29-1.51)	0.33
PC(22:6(4Z,7Z,10Z,13Z,16Z,19Z)/18:1(11Z))	0.72 (0.49-1.06)	0.10	0.45 (0.26-0.78)	<0.05	0.63 (0.26-1.51)	0.30
LysoPC(18:3(6Z,9Z,12Z))*	2.09 (1.09-4.01)	<0.05	3.92 (2.35-6.54)	<0.05	4.89 (1.57-15.23)	0.02
Octanoylglucuronide	0.03 (0.01-0.11)	<0.05	0.02 (0.003-0.11)	<0.05	0.65 (0.03-14.08)	0.35
LysoPC(20:4(5Z,8Z,11Z,14Z))	0.01 (0.001-0.19)	<0.05	0.01 (0.001-0.20)	<0.05	0.20 (0.003-13.33)	0.94
Phenylalanyl-Tryptophan	0.02 (0.003-0.09)	<0.05	0.02 (0.002-0.11)	<0.05	0.09 (0.001-8.10)	0.61
PS(22:6/22:4)	0.01 (0.001-0.07)	<0.05	0.00 (0.001-0.12)	<0.05	0.11 (0.002-6.05)	0.26
Cholesterol glucuronide	0.01 (0.001-0.10)	<0.05	0.01 (0.001-0.14)	<0.05	0.11 (0.01-1.21)	0.28
LysoPC(18:2(9Z,12Z))*	0.19 (0.10-0.37)	<0.05	0.20 (0.10-0.40)	<0.05	0.11 (0.03-0.45)	<0.05
LysoPC(22:0)*	0.11 (0.05-0.25)	<0.05	0.12 (0.05-0.28)	<0.05	0.21 (0.08-0.57)	<0.05
PE(P-18:1(9Z)/18:1(9Z))*	0.43 (0.28-0.67)	<0.05	0.44 (0.27-0.72)	<0.05	0.11 (0.03-0.42)	<0.05
Oleic acid	1.43 (0.88-2.33)	0.15	1.42 (0.85-2.40)	0.18	2.70 (0.99-7.32)	0.05
N6-Methyladenosine	2.32 (2.01-2.68)	<0.05	2.45 (1.96-3.06)	<0.05	2.51 (0.08-78.75)	0.75
2-Ketobutyric acid*	5.61 (2.16-14.59)	<0.05	5.21 (2.00-13.56)	<0.05	13.44 (2.56-70.69)	<0.05
**Urine metabolites**						
Vinylacetylglycine	0.81 (0.57-1.16)	0.25	0.74 (0.47-1.15)	0.17	0.39 (0.17-0.92)	0.03
Allysine	0.84 (0.58-1.21)	0.34	0.96 (0.65-1.42)	0.83	0.37 (0.13-1.00)	0.05
N6-Acetyl-L-lysine*	5.03 (1.41-17.92)	0.01	6.99 (1.74-28.05)	0.01	9.77 (1.19-80.26)	0.03
Corticosterone*	0.44 (0.30-0.66)	<0.05	0.41 (0.25-0.68)	<0.05	0.36 (0.16-0.80)	0.01

Supplemental Table S1 Continued

	Crude model		Adjust I		Adjust II	
	**OR (95%CI)**	***p value***	**OR (95%CI)**	***p value***	**OR (95%CI)**	***p value***
Erythronic acid*	0.44 (0.29-0.67)	<0.05	0.36 (0.21-0.60)	<0.05	0.21 (0.07-0.59)	<0.05
Cortisol	0.58 (0.40-0.85)	<0.05	0.57 (0.37-0.88)	0.01	0.58 (0.25-1.35)	0.20
Uracil*	0.29 (0.17-0.51)	<0.05	0.18 (0.09-0.39)	<0.05	0.05 (0.01-0.31)	<0.05
Argininosuccinic acid*	0.43 (0.28-0.65)	<0.05	0.41 (0.25-0.67)	<0.05	0.27 (0.10-0.76)	0.01
Xanthurenic acid*	0.36 (0.23-0.55)	<0.05	0.38 (0.23-0.62)	<0.05	0.41 (0.21-0.81)	0.01
Caprylic acid	0.00 (0.001-0.04)	<0.05	0.30 (0.004-20.19)	0.99	0.07 (0.001-4.90)	0.29
N4-Acetylcytidine*	0.27 (0.16-0.46)	<0.05	0.30 (0.17-0.54)	<0.05	0.31 (0.13-0.74)	0.01
Suberylglycine	0.58 (0.40-0.84)	<0.05	0.46 (0.29-0.73)	<0.05	0.53 (0.27-1.04)	0.06
Cyclic GMP*	2.63 (1.27-5.44)	0.01	3.05 (1.36-6.88)	0.01	12.22 (2.00-74.71)	0.01

Crude Model, an unadjusted model; Adjust I, a minimally adjusted model that includes the covariates age, sex and BMI; Adjust II, a fully adjusted model that includes the covariates age, sex, BMI, TG, TC, HDL-C, LDL-C, hypertension, diabetes, and smoking. OR, per-standard deviation odds ratio. 95%CI, 95% confidence interval. *$p < 0.05$, remained statistical significantly in crude and adjusted model.

Supplemental Table S2 The correlation between the four potential metabolic biomarkers and ACS phenotype

	2-Ketobutyric acid		LysoPC(18:2(9Z,12Z))		Argininosuccinic acid		Cyclic GMP	
	**rho**	***p value***	**rho**	***p value***	**rho**	***p value***	**rho**	***p value***
**cTnI**	0.50	<0.05	-0.48	<0.05	-0.42	<0.05	0.29	<0.001
**NT-proBNP**	0.42	<0.05	-0.43	<0.05	-0.39	<0.05	0.31	<0.001
**hs-CRP**	0.42	<0.05	-0.58	<0.05	-0.31	<0.001	0.31	<0.001
**LVEF**	-0.38	<0.05	0.23	<0.01	0.33	<0.05	-0.22	0.01
**SYNTAXI**	0.24	<0.01	-0.40	<0.05	-0.32	<0.001	0.19	0.02
**SYNTAXII**	0.28	<0.001	-0.41	<0.05	-0.33	<0.05	0.09	0.28
**Gensini**	0.23	<0.001	-0.36	<0.05	-0.31	<0.001	0.21	0.01
**No. of SV**	0.17	0.04	-0.40	<0.05	-0.22	0.01	0.18	0.03

Supplemental Table S3 The correlation between 15 serum and 13 urine metabolites

	2-Ketobutyric acid		Cholesterol glucuronide		LysoPC (18:2(9Z,12Z))		LysoPC (18:3(6Z,9Z,12Z))		LysoPC(20:4 (5Z,8Z,11Z,14Z))	
	**rho**	***p value***	**rho**	***p value***	**rho**	***p value***	**rho**	***p value***	**rho**	***p value***
**Allysine**	-0.26	1.44E-03	0.19	2.27E-02	0.34	2.91E-05	0.06	4.57E-01	0.15	7.04E-02
**Argininosuccinic acid**	-0.26	1.17E-03	0.19	2.29E-02	0.19	1.78E-02	-0.13	1.17E-01	0.22	5.76E-03
**Caprylic acid**	-0.28	4.82E-04	0.41	1.75E-07	0.38	2.02E-06	-0.41	3.43E-07	0.39	8.53E-07
**Corticosterone**	-0.11	1.89E-01	0.26	1.42E-03	0.07	3.84E-01	-0.30	2.11E-04	0.28	5.18E-04
**Cortisol**	0.00	9.74E-01	0.14	9.79E-02	-0.07	3.92E-01	-0.20	1.42E-02	0.16	5.73E-02
**Cyclic GMP**	0.17	3.80E-02	-0.22	6.44E-03	-0.20	1.33E-02	0.13	1.07E-01	-0.13	1.05E-01
**Erythronic acid**	0.02	7.84E-01	0.20	1.37E-02	0.01	8.62E-01	-0.26	1.28E-03	0.19	1.82E-02
**N4-Acetylcytidine**	-0.27	9.32E-04	0.28	4.33E-04	0.22	5.97E-03	-0.29	2.92E-04	0.27	8.04E-04
**N6-Acetyl-L-lysine**	-0.02	8.30E-01	-0.17	3.31E-02	0.05	5.30E-01	0.33	4.88E-05	-0.05	5.39E-01
**Suberylglycine**	-0.20	1.24E-02	0.14	7.79E-02	0.25	1.72E-03	-0.11	1.64E-01	0.20	1.22E-02
**Uracil**	-0.31	1.51E-04	0.28	6.16E-04	0.35	9.94E-06	-0.27	1.03E-03	0.39	8.30E-07
**Vinylacetylglycine**	-0.13	1.14E-01	0.04	6.46E-01	0.16	5.01E-02	0.01	9.49E-01	0.18	2.91E-02
**Xanthurenic acid**	-0.13	1.07E-01	0.34	3.03E-05	0.26	1.59E-03	-0.24	2.96E-03	0.34	2.32E-05

Supplemental Table S3 Continued

	LysoPC(22:0)		N6-Methyladenosine		Octanoylglucuronide		Oleic acid		PC(22:6(4Z,7Z,10Z,13Z,16Z,19Z)/18:1(11Z))	
	rho	*p value*	rho	*p value*	rho	*p value*	rho	*p value*	rho	*p value*
**Allysine**	0.33	3.44E-05	-0.26	1.26E-03	0.45	9.12E-09	-0.38	2.34E-06	0.14	9.68E-02
**Argininosuccinic acid**	0.29	3.36E-04	-0.42	8.13E-08	0.30	1.61E-04	-0.05	5.64E-01	0.25	1.86E-03
**Caprylic acid**	0.54	0.00E+00	-0.39	8.37E-07	0.53	0.00E+00	-0.14	9.64E-02	0.27	1.01E-03
**Corticosterone**	0.08	3.60E-01	-0.38	2.49E-06	0.22	8.13E-03	0.06	4.85E-01	-0.10	2.17E-01
**Cortisol**	0.05	5.43E-01	-0.23	4.72E-03	0.17	3.52E-02	0.22	6.66E-03	0.18	2.90E-02
**Cyclic GMP**	-0.30	2.53E-04	0.13	1.16E-01	-0.28	4.78E-04	0.13	1.25E-01	-0.16	4.77E-02
**Erythronic acid**	0.07	3.70E-01	-0.25	1.84E-03	0.10	2.22E-01	0.19	1.81E-02	0.13	1.05E-01
**N4-Acetylcytidine**	0.22	6.43E-03	-0.43	4.56E-08	0.34	1.79E-05	-0.05	5.53E-01	0.04	5.86E-01
**N6-Acetyl-L-lysine**	-0.11	1.66E-01	0.03	7.31E-01	-0.13	1.06E-01	-0.18	2.92E-02	-0.05	5.19E-01
**Suberylglycine**	0.30	1.68E-04	-0.28	6.85E-04	0.31	1.56E-04	-0.25	2.57E-03	0.20	1.38E-02
**Uracil**	0.35	9.89E-06	-0.53	0.00E+00	0.34	2.12E-05	-0.16	4.66E-02	0.16	4.62E-02
**Vinylacetylglycine**	0.26	1.10E-03	-0.14	9.35E-02	0.17	3.50E-02	-0.29	2.65E-04	0.11	1.97E-01
**Xanthurenic acid**	0.22	7.25E-03	-0.45	1.66E-08	0.28	4.31E-04	-0.09	2.92E-01	0.05	5.54E-01

Supplemental Table S3 Continued

	PE(P-18:1(9Z)/18:1(9Z))		Phenylalanyl-Tryptophan		Proline betaine		PS (22:6/22:4)		SM(d18:1/20:0)	
	rho	*p value*	rho	*p value*	rho	*p value*	rho	*p value*	rho	*p value*
**Allysine**	0.34	2.17E-05	0.29	3.22E-04	0.08	3.02E-01	0.10	2.45E-01	0.18	2.54E-02
**Argininosuccinic acid**	0.21	1.14E-02	0.38	1.47E-06	0.06	4.87E-01	0.27	9.16E-04	0.18	2.48E-02
**Caprylic acid**	0.33	4.10E-05	0.58	0.00E+00	0.19	1.80E-02	0.44	1.99E-08	0.16	5.22E-02
**Corticosterone**	0.03	7.04E-01	0.29	2.64E-04	-0.11	1.86E-01	0.22	6.88E-03	-0.13	1.02E-01
**Cortisol**	-0.07	3.70E-01	0.23	5.72E-03	-0.14	8.07E-02	0.20	1.22E-02	-0.12	1.43E-01
**Cyclic GMP**	-0.27	9.75E-04	0.03	6.95E-01	-0.14	8.92E-02	-0.13	1.05E-01	-0.18	3.07E-02
**Erythronic acid**	-0.07	3.73E-01	0.28	5.06E-04	-0.06	4.60E-01	0.31	1.34E-04	-0.12	1.49E-01
**N4-Acetylcytidine**	0.15	6.82E-02	0.30	2.43E-04	-0.01	8.60E-01	0.34	2.84E-05	-0.01	9.23E-01
**N6-Acetyl-L-lysine**	0.01	8.81E-01	-0.09	2.62E-01	-0.02	8.53E-01	-0.24	3.13E-03	0.15	7.47E-02
**Suberylglycine**	0.14	9.69E-02	0.26	1.55E-03	0.16	4.73E-02	0.15	5.83E-02	0.02	8.31E-01
**Uracil**	0.21	8.61E-03	0.44	1.89E-08	0.07	4.05E-01	0.34	3.07E-05	-0.01	9.43E-01
**Vinylacetylglycine**	0.20	1.62E-02	0.13	1.03E-01	0.71	0.00E+00	0.08	3.05E-01	0.25	1.89E-03
**Xanthurenic acid**	0.05	5.13E-01	0.31	1.26E-04	0.02	8.21E-01	0.27	8.53E-04	-0.05	5.73E-01
